# In-depth comparison of the metabolic and pharmacokinetic behaviour of the structurally related synthetic cannabinoids AMB-FUBINACA and AMB-CHMICA in rats

**DOI:** 10.1038/s42003-022-03113-5

**Published:** 2022-02-24

**Authors:** David Fabregat-Safont, María Mata-Pesquera, Manuela Barneo-Muñoz, Ferran Martinez-Garcia, Marie Mardal, Anders B. Davidsen, Juan V. Sancho, Félix Hernández, María Ibáñez

**Affiliations:** 1 Environmental and Public Health Analytical Chemistry, Research Institute for Pesticides and Water (IUPA), University Jaume I, Avda. Sos Baynat s/n, 12071 Castellón, Spain; 2 Predepartmental Unit of Medicine, Unitat Mixta de Neuroanatomia Funcional NeuroFun-UVEG-UJI, University Jaume I, Castellón, Spain; 3 Section of Forensic Chemistry, Department of Forensic Medicine, University of Copenhagen, Copenhagen, Denmark

**Keywords:** Biomarkers, Toxicology

## Abstract

Synthetic cannabinoids receptor agonists (SCRAs) are often almost completely metabolised, and hence their pharmacokinetics should be carefully evaluated for determining the most adequate biomarker in toxicological analysis. Two structurally related SCRAs, AMB-FUBINACA and AMB-CHMICA, were selected to evaluate their in vivo metabolism and pharmacokinetics using male Sprague-Dawley rats. Brain, liver, kidney, blood (serum) and urine samples were collected at different times to assess the differences in metabolism, metabolic reactions, tissue distribution and excretion. Both compounds experimented *O*-demethyl reaction, which occurred more rapidly for AMB-FUBINACA. The parent compounds and *O*-demethyl metabolites were highly bioaccumulated in liver, and were still detected in this tissue 48 h after injection. The different indazole/indole *N*-functionalisation produced diverse metabolic reactions in this moiety and thus, different urinary metabolites were formed. Out of the two compounds, AMB-FUBINACA seemed to easily cross the blood-brain barrier, presenting higher brain/serum concentrations ratio than AMB-CHMICA.

## Abstract (web summary)

Synthetic cannabinoids are amongst the most widely used psychoactive drugs which are tightly controlled by government agencies around the world. Here, pharmacokinetics of two synthetic cannabinoids in rats are evaluated along with their metabolites and tissue distribution, aiding in identifying distinct biomarkers that reflect the consumption of synthetic cannabinoids based on the tissue.

## Introduction

Synthetic cathinones and synthetic cannabinoids are among the most consumed psychoactive substances (NPS) in Europe^1^. In 2019, synthetic cannabinoids and cathinones accounted for almost 60% of the number of seizures reported by EU Member States. Additionally, 46 NPS were reported for the first time in Europe during 2020, reaching approximately a total of 830 NPS the number of compounds being monitored by the European Monitoring Centre for Drugs and Drug Addiction (EMCDDA)^1^. In fact, the number of intoxication cases related to synthetic cannabinoid receptor agonists (SCRAs, commonly named as synthetic cannabinoids) reported by the European Drug Emergencies Network has increased in the last years, as illustrated by an outbreak of over 20 deaths related to the synthetic cannabinoid 4F-MDMB-BICA in 2020^1^.

In the last years, many scientific publications dealing with SCRAs have been published, reflecting the increasing interest in these compounds. The fast changes in the psychoactive market promote the emergence of novel compounds not previously reported, that should be analytically characterised^2–4^. The continuous moving NPS market makes complicated their monitoring, legal regulation, and the study of their toxicological effects and metabolism. An important aspect on SCRAs research is the evaluation of their potency, in terms of affinity to the cannabinoid receptors CB1 and CB2^5–7^, as well as their potential toxicity^8–11^. The information related to potency and toxicity is used to assess the health risks associated to the use of these substances, and to propose medical treatment for intoxication cases related to SCRAs consumption. Updated analytical methodologies are required for the identification and quantification of SCRAs in authentic human samples, such as blood and urine^8,12,13^. Indeed, SCRA metabolites should also be included in the analytical methods due to the fast metabolism of these groups of NPS. This makes that the unaltered molecule is not commonly detected in urine samples^14,15^, and therefore the monitoring must be directed towards the main metabolites (consumption biomarkers)^16^.

There are different approaches for evaluating the metabolism of SCRAs, such as in vitro models like incubation with liver microsomes^17,18^ or pooled hepatocytes^19,20^, in vivo experiments using rats or other animals^15,21^, analysis of authentic human samples from intoxication cases^22,23^, or in silico prediction tools^24^. Although the most accurate biomarkers are obtained from metabolite detection in human samples, the availability of these matrices is limited to intoxication cases or clinical trials. In intoxication cases, the pharmacokinetics and excretion of these metabolites cannot be fully evaluated in many cases, because of the first sampling is performed during clinical cares after intoxication diagnosis. At this point, in vivo experiments in model animals provide information about the metabolism of a certain substance, as well as the distribution of the parent compound and/or metabolites in different tissues along the time, including the excretion of these compounds^25^.

Metabolism studies of SCRAs indicate that these compounds present similar biotransformations related to their moieties^24^. Nevertheless, similar biotransformations may not lead to the same pharmacokinetics, including tissue distribution and excretion. For this reason, the aim of this work was to evaluate the in vivo metabolism, tissue distribution, and excretion of two structurally related SCRAs. AMB-FUBINACA (also known as MMB-FUBINACA) and AMB-CHMICA (MMB-CHMICA) were selected as model compounds, and male Sprague-Dawley rats were used as animal model. The SCRAs selection was based on their structural similarities (presence of a methyl valinate moiety), which yield the same main metabolic reaction: *O*-demethylation in the methyl ester moiety^26,27^. For both compounds, their metabolites were elucidated and analysed in different tissues, such as brain, liver, and kidney, as well as in blood and urine, collected at distinct sampling times. Samples were analysed by ultra-high performance liquid chromatography coupled to high-resolution mass spectrometry (UHPLC-HRMS) working on data-independent acquisition (DIA) mode, a technique that has proven its great potential in NPS research, including metabolite elucidation^28^. Additionally, the distribution and pharmacokinetics of the parent compounds and metabolites in the different tissues were investigated. Finally, the most suitable biomarkers of consumption have been proposed depending on the type of sample analysed.

## Results and discussion

### Metabolite elucidation based on HRMS data

Metabolites detected by the described strategies were elucidated based on the calculated elemental composition of the metabolites (low energy spectra, LE) and on the observed fragmentation (high energy spectra, HE). The biotransformation was located by comparing the fragmentation spectra of metabolite and parent compound, identifying the unaltered moieties and thus placing the corresponding biotransformation.

**Fig. 1:**
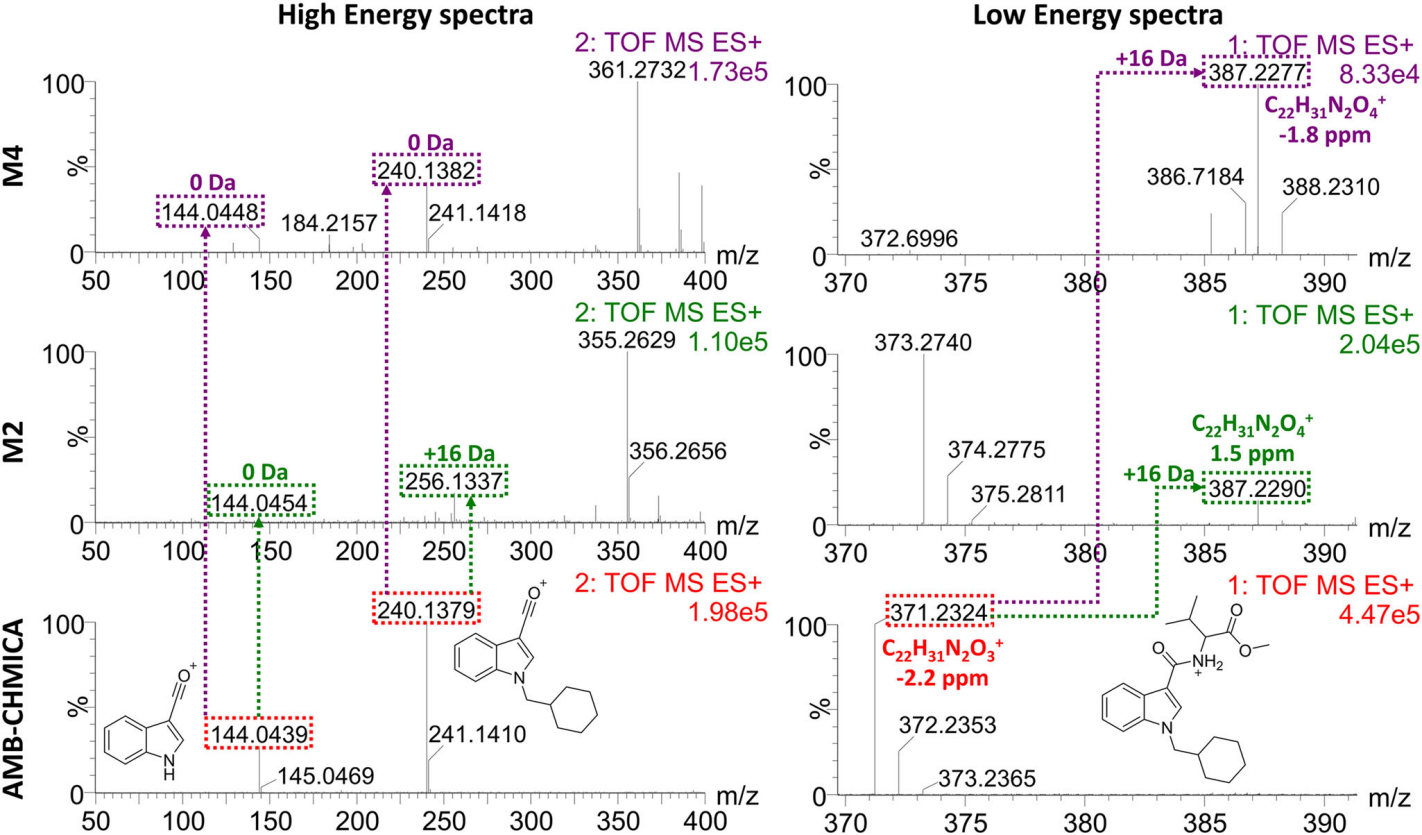
Elucidation of hydroxylated AMB-CHMICA metabolites by UHPLC-HRMS. Low energy and high energy spectra for parent compound, metabolite M2, and metabolite M4 are shown. Red text and dashed lines refer to AMB-CHMICA, green to metabolite M2, and purple to metabolite M4. Mass errors have been calculated in parts per million (ppm), as follows: ${{{{{\rm{ppm}}}}}}=\frac{{{{{{\rm{accurate}}}}}}\; {{{{{\rm{mass}}}}}}-{{{{{\rm{exact}}}}}}\; {{{{{\rm{mass}}}}}}}{{{{{{\rm{exact}}}}}}\; {{{{{\rm{mass}}}}}}}\cdot {10}^{6}$.

As illustrative example, Fig. 1 shows the elucidation of two hydroxylated metabolites of AMB-CHMICA. The fragment at *m/z* 144, observed in the parent compound, was also present in the HE spectra of the two metabolites, therefore discarding the hydroxylation in the indole ring. In the compound M4, the fragment *m/z* 240 was also observed, suggesting the biotransformation to take place in the methyl valinate moiety, surely in the isopropyl group. In the case of M2, the fragment ion at *m/z* 256, corresponding to the *N*-cyclohexylmethyl indole, presented a +16 Da shift respect the AMB-CHMICA fragment. So, the hydroxylation occurred in this part of the molecule, specifically in the cyclohexylmethyl moiety based on the presence of fragment ion at *m/z* 144.

In parallel to the evaluation of the fragment ions observed, extracted ion chromatograms (EICs) were extracted to search for additional metabolites on the basis of common fragmentation pathways.

Summarising, only phase I metabolites were elucidated for both compounds (all of them detected in positive ionisation mode), some of which had not been reported in previous in vitro metabolism studies.

### AMB-FUBINACA

**Fig. 2:**
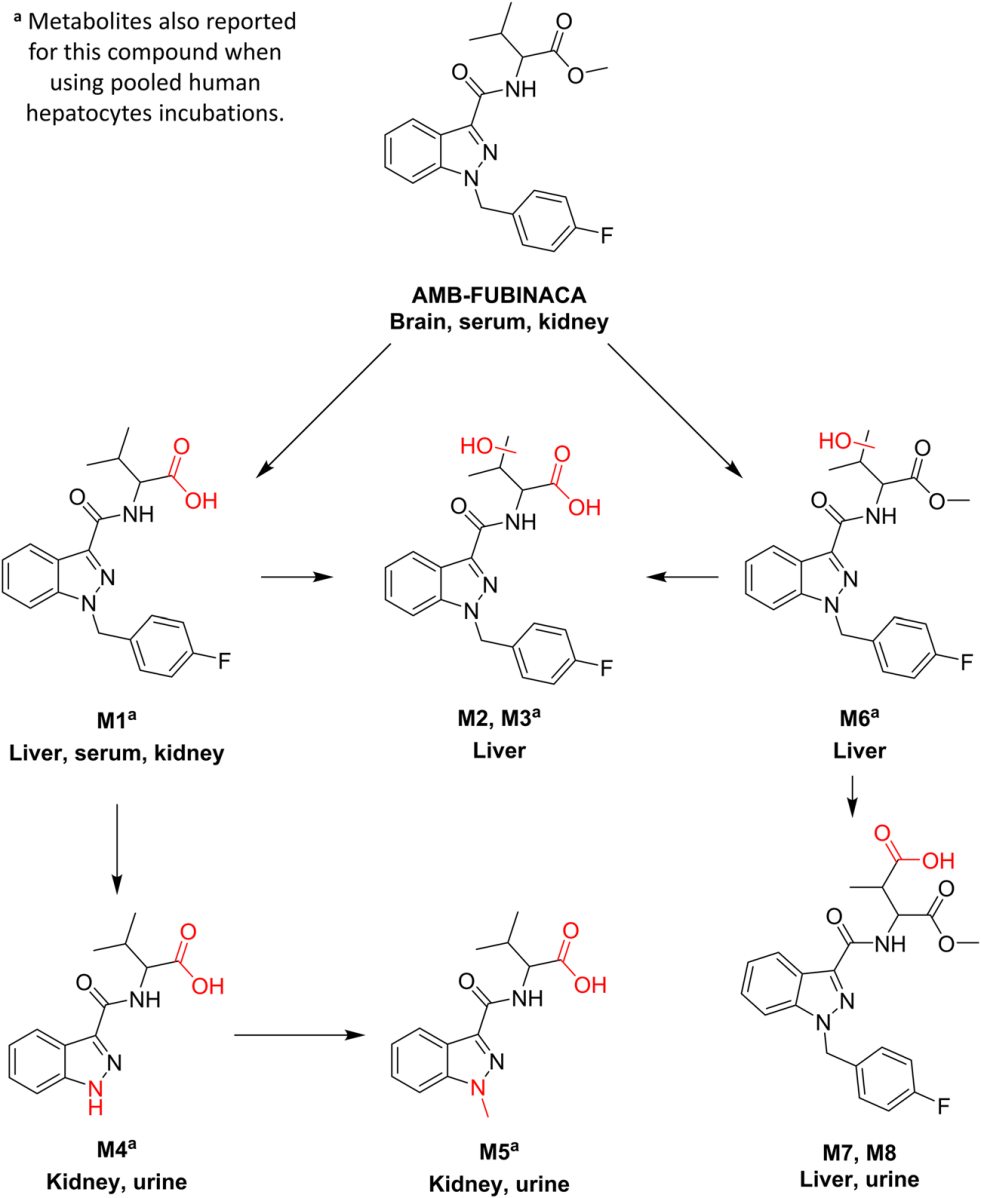
Proposed metabolic pathway for AMB-FUBINACA. Red moieties in compound structures indicate biotransformations. Tissue names indicate the prevalence of the metabolite.

Up to 8 phase I metabolites were detected for AMB-FUBINACA after evaluating the 5 studied matrices, as a result of hydroxylation, oxidation, and dealkylation (and combination) processes. Figure 2 shows the proposed metabolic pathway for this compound in male Sprague-Dawley rats, indicating also those metabolites detected by incubation with pooled human hepatocytes^19^. The information about chromatographic retention time, accurate-mass fragmentation, elemental composition, and mass error of AMB-FUBINACA metabolites are shown in Table 1, as well as the LE and HE spectra (Figs. S1–S10 of Supplementary Information).

**Table 1:** UHPLC-HRMS detection of AMB-FUBINACA and its metabolites.

Compound	RT (min)	[M + H]^+^ (*m/z*)	Elemental composition	Mass error (ppm)	Fragment (*m/z*)	Elemental composition	Mass error (ppm)
AMB-FUBINACA	9.16	384.1717	C_21_H_23_FN_3_O_3_^+^	−1.6	324.1508	C_19_H_19_FN_3_O^+^	−1.2
					253.0769	C_15_H_10_FN_2_O^+^	−3.2
					109.0448	C_7_H_6_F^+^	−5.5
M1	7.92	370.1565	C_20_H_21_FN_3_O_3_^+^	−0.5	324.1505	C_19_H_19_FN_3_O^+^	−2.2
(*O*-demethyl)					253.0774	C_15_H_10_FN_2_O^+^	−1.2
					109.0450	C_7_H_6_F^+^	−3.7
M2	6.76	386.1515	C_20_H_21_FN_3_O_4_^+^	−0.3	253.0761	C_15_H_10_FN_2_O^+^	−1.6
(*O*-demethyl, OH-isopropyl)					109.0448	C_7_H_6_F^+^	−5.5
M3	6.61	386.1509	C_20_H_21_FN_3_O_4_^+^	−1.8	253.0766	C_15_H_10_FN_2_O^+^	−4.3
(*O*-demethyl, OH-isopropyl)					109.0457	C_7_H_6_F^+^	2.8
M4	5.35	262.1191	C_13_H_16_N_3_O_3_^+^	−0.4	216.1135	C_12_H_14_N_3_O^+^	−0.9
(*O*-demethyl, *N*-dealkyl)					145.0396	C_8_H_5_N_2_O^+^	−4.1
M5	6.13	276.1346	C_14_H_18_N_3_O_3_^+^	−0.7	230.1283	C_13_H_16_N_3_O^+^	−4.3
(*O*-demethyl, *N*-dealkyl, *N*-methyl)					159.0553	C_9_H_7_N_2_O^+^	−3.1
M6	7.76	400.1663	C_21_H_23_FN_3_O_4_^+^	−2.5	350.1307	C_20_H_17_FN_3_O_2_^+^	0.6
(OH-isopropyl)					253.0773	C_15_H_10_FN_2_O^+^	1.6
					109.0442	C_7_H_6_F^+^	−11.0
M7	7.52	414.1457	C_21_H_21_FN_3_O_5_^+^	−1.9	253.0763	C_15_H_10_FN_2_O^+^	−5.5
(COOH-isopropyl)					109.0446	C_7_H_6_F^+^	−7.3
M8	7.42	414.1452	C_21_H_21_FN_3_O_5_^+^	−3.1	253.0766	C_15_H_10_FN_2_O^+^	−4.3
(COOH-isopropyl)					109.0444	C_7_H_6_F^+^	−9.2

The first AMB-FUBINACA biotransformations found were *O*-demethylation (M1) and OH-isopropyl (M6) in the methyl valinate moiety, as well as the combination of both biotransformations, similarly to previously reported SCRAs^29^. The presence of two resolved chromatographic peaks corresponding to *O*-demethylation+OH-isopropyl suggests that both methyl moieties of the isopropyl were hydroxylated, giving two isomeric metabolites (M2 and M3), or maybe one of these metabolites was hydroxylated in the tertiary carbon of the isopropyl group. In the case of M6, only one peak was observed with an OH-isopropyl, indicating that only one metabolite was obtained or maybe the two isomeric compounds were not chromatographically resolved.

After *O*-demethylation, *N*-dealkylation was found (M4), followed by an *N*-methylation (M5). These two metabolites were also observed using pooled human hepatocytes^19^. The *N*-dealkylation has been reported for other SCRAs^23,30^, but no information was found for the *N*-methylated metabolite except for the in vitro study^19^. In that case, this compound might be an artefact produced by the presence of methanol traces in the incubation media^19,31^. Nevertheless, methanol was not used in the present study, as the compounds were dissolved in saline containing ethanol, and sample treatment and chromatographic separation were performed using acetonitrile. All these facts suggest that *N*-methylation in the indazole ring had been produced by metabolic processes.

Two additional metabolites (M7 and M8) were observed after detecting M6, being carboxylated in the isopropyl moiety (Fig. 2). The presence of these two isomeric compounds suggest that two hydroxylation points also occur for M6 but these metabolites were not chromatographically separated, as previously speculated. The isopropyl carboxylation was not described in the in vitro study, probably due to the differences between in vitro and in vivo models. Except for these metabolites, the remaining detected compounds had also been observed in the in vitro model^19^, increasing the confidence on the proposed metabolic pathway for AMB-FUBINACA (Fig. 2).

All of the previously described metabolites were detected in positive ionisation mode, as well as in negative mode for those metabolites with acid moieties, such as M1, M7, and M8. Nevertheless, the instrumental response observed in negative ionisation was lower than in positive and, for that, data evaluation was performed only in positive mode.

Once the AMB-FUBINACA metabolites were elucidated in the study matrices, data were reprocessed using a target method for the determination of the analytical responses of all these compounds in the whole set of samples, in order to assess their distribution and pharmacokinetics. Analytical responses obtained for AMB-FUBINACA and its metabolites in all the analysed matrices are available in Table S1. In the case of urine, some samples were not available, as the rats did not excrete enough urine for performing sample treatment.

It can be noticed the high abundance of unaltered AMB-FUBINACA in brain samples at 15 min, whereas no additional metabolites were detected in this tissue. AMB-FUBINACA was also detected in kidney, and at lesser extent in serum and liver samples, but not in urine samples. The main metabolite M1 presented the highest response in liver, whereas M4 and M5 were the major urinary metabolites. M1 was also detected in liver and serum samples collected 48 h after injection, and M3, M4, M5, M7, and M8 were found in 24 h urine samples. No additional metabolites were found in the 48 h sample used as control.

### AMB-CHMICA

Nine phase I metabolites, produced by hydroxylation, dealkylation, and different combinations, were identified in AMB-CHMICA (Fig. 3). Four of these metabolites had also been reported after pooled human hepatocytes incubation^27^. The full analytical data of the elucidated compounds are presented in Table 2, and the LE and HE spectra are shown in Figs. S10–S19.

**Fig. 3:**
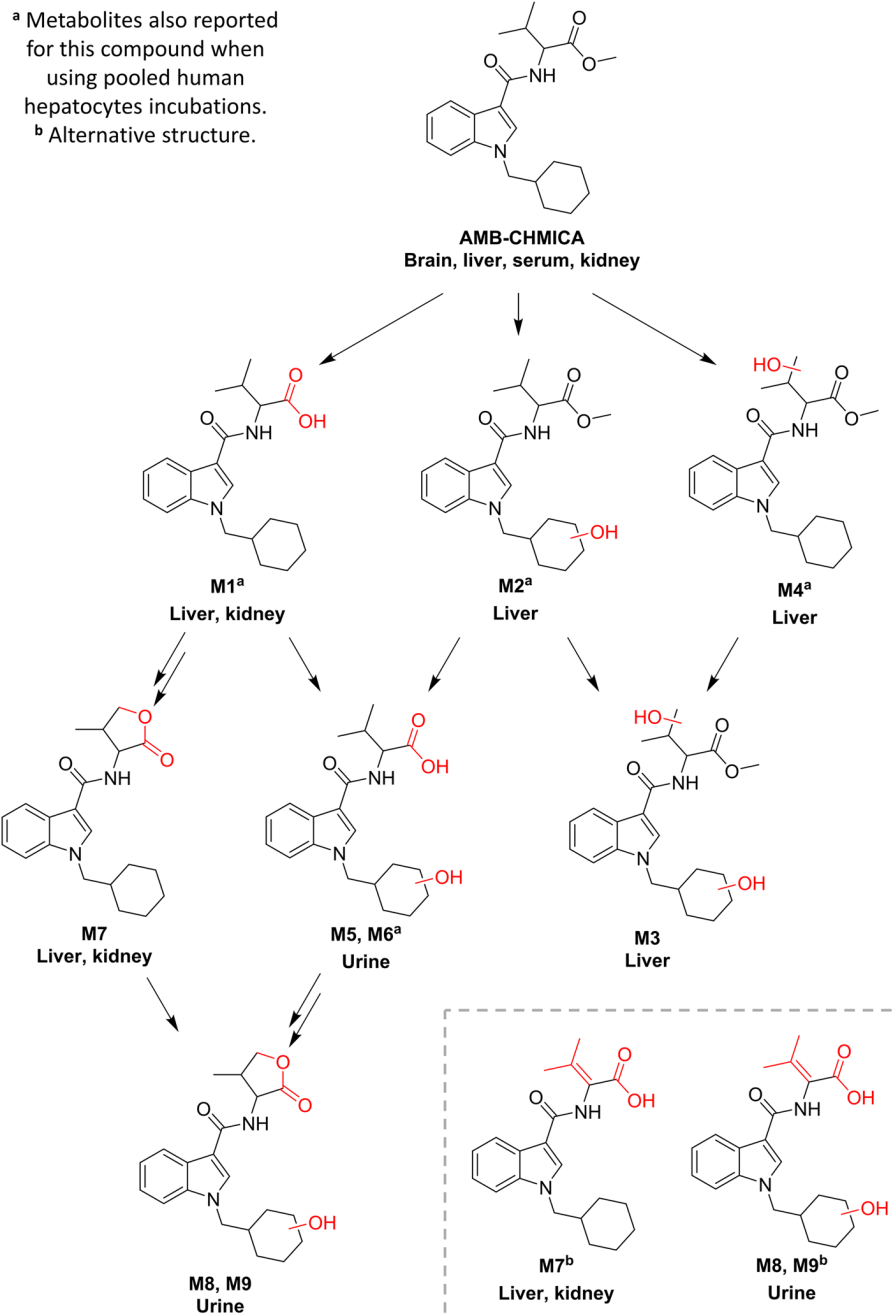
Proposed metabolic pathway for AMB-CHMICA. Red moieties in compound structures indicate biotransformations. Tissue names indicate prevalence of the metabolite.

**Table 2:** UHPLC-HRMS detection of AMB-CHMICA and its metabolites.

Compound	RT (min)	[M + H]^+^ (*m/z*)	Elemental composition	Mass error (ppm)	Fragment (*m/z*)	Elemental composition	Mass error (ppm)
AMB-CHMICA	9.80	371.2327	C_22_H_31_N_2_O_3_^+^	−2.2	240.1382	C_16_H_18_NO^+^	−2.5
					144.0443	C_9_H_6_NO^+^	−4.2
					97.1014	C_7_H_13_^+^	−3.1
M1	8.68	357.2169	C_21_H_29_N_2_O_3_^+^	−2.5	240.1383	C_16_H_18_NO^+^	−2.1
(*O*-demethyl)					144.0439	C_9_H_6_NO^+^	−6.9
M2	7.24	387.2290	C_22_H_31_N_2_O_4_^+^	1.5	256.1336	C_16_H_18_NO_2_^+^	−0.8
(OH-cyclohexyl)					144.0455	C_9_H_6_NO^+^	4.2
M3	6.88	403.2236	C_22_H_31_N_2_O_5_^+^	0.7	256.1320	C_16_H_18_NO_2_^+^	−7.0
(OH-cyclohexyl, OH-isopropyl)					144.0446	C_9_H_6_NO^+^	−2.1
M4	8.72	387.2277	C_22_H_31_N_2_O_4_^+^	−1.8	240.1382	C_16_H_18_NO^+^	−2.5
(OH-isopropyl)					144.0448	C_9_H_6_NO^+^	−0.7
M5	6.20	373.2118	C_21_H_29_N_2_O_4_^+^	−2.4	256.1342	C_16_H_18_NO_2_^+^	1.6
(*O*-demethyl, OH-cyclohexyl)					144.0453	C_9_H_6_NO^+^	2.8
M6	6.02	373.2117	C_21_H_29_N_2_O_4_^+^	−2.7	256.1336	C_16_H_18_NO_2_^+^	−0.8
(*O*-demethyl, OH-cyclohexyl)					144.0453	C_9_H_6_NO^+^	2.8
M7	8.50	355.2020	C_21_H_27_N_2_O_3_^+^	−0.6	240.1387	C_16_H_18_NO^+^	−0.4
(isopropyl lactone)					144.0458	C_9_H_6_NO^+^	6.2
M8	5.75	371.1977	C_21_H_27_N_2_O_4_^+^	1.6	256.1339	C_16_H_18_NO_2_^+^	0.4
(isopropyl lactone, OH-cyclohexyl)					144.0456	C_9_H_6_NO^+^	4.9
M9	5.97	371.1970	C_21_H_27_N_2_O_4_^+^	−0.3	256.1333	C_16_H_18_NO_2_^+^	−2.0
(isopropyl lactone, OH-cyclohexyl)					144.0456	C_9_H_6_NO^+^	4.9

The first metabolites detected for AMB-CHMICA were *O*-demethylation (M1) and hydroxylation in the isopropyl (M4) of the methyl valinate moiety, as well as in the cyclohexylmethyl (M2). After that, M3 was found, corresponding to OH-isopropyl+OH-cyclohexylmethyl, while M5 and M6 resulted from *O*-demethylation+OH-cyclohexylmethyl. 4 of these 5 metabolites had also been observed in human hepatocyte incubations^27^, although some differences were found. Regarding M2 (OH-cyclohexylmethyl), only one compound was observed in the present study, whereas 4 isomers of this metabolite were found in in vitro experiments^27^. The compound M3 was not observed in the human hepatocytes incubation, while additional biotransformations such as indole *N*-dealkylation, OH-indole, diOH-cyclohexylmethyl, and their combinations, were not found in the Sprague-Dawley rat samples but were observed in the in vitro model.

Nevertheless, an additional biotransformation not observed in in vitro experiments was found. M7 (Fig. 3 and Table 2) presented fragment ions at *m/z* 144 and 240, suggesting that the biotransformation was placed on the methyl valinate moiety. This compound had a −16 Da shift respect to AMB-CHMICA (corresponding to a CH_4_ loss), −2 Da respect M1 (H_2_ loss) and −32 Da respect M4 (CH_4_O loss). It must be taken into account that compounds with carboxylic acids and hydroxyls, such as atorvastatin, can produce lactone metabolites^32^. So, M7 was proposed to be a lactone originated from a non-detected metabolite corresponding to the isopropyl hydroxylation of M1. Lactonization is produced when the hydroxyl group of an alkyl chain near a carboxylic acid produces a nucleophilic substitution in the carbonyl, obtaining a lactone and a water molecule loss^32^. This biotransformation has been reported for the closely analogue ADB-CHMINACA when using human hepatocytes^33^. Another possibility is the presence of an insaturation in the valinate moiety produced by a dehydrogenation catalysed by cytochrome P450, as minor dehydrogenated metabolites had been reported together with major hydroxylated metabolites produced by this enzyme^34^. In any case, the structure of this metabolite could not be assured based on the available fragmentation, and the analytical reference standard should be synthetised for unequivocal identification.

M7 was then metabolised through OH-cyclohexylmethyl, obtaining two isomeric metabolites (M8 and M9), justifying the hydroxylated moiety based on accurate-mass fragmentation. The detection of these isomers is in accordance to the results obtained by human hepatocytes incubation as previously commented, in spite of the no detection of positional isomers for M2. As shown in Table 2, M8 and M9 had similar chromatographic retention time and thus, it is possible that potential isomers of M2 were not chromatographically resolved. Up to 4 of the AMB-CHMICA metabolites detected in Sprague-Dawley rat samples were also reported using pooled human hepatocytes^27^.

Other metabolites reported in vitro for AMB-CHIMCA, such as OH-indazole and OH-cyclohexane, were not observed in vivo^27^.

AMB-CHMICA and its metabolites analytical responses were obtained after data reprocessing (Table S2). AMB-CHMICA was detected in brain samples at 15 and 30 min, as well as traces of M4 and M7 metabolites. In the case of liver samples, AMB-CHMICA was the major compound at low sampling times together with M4 and three minor metabolites, while at high sampling times M1 became the major compound in liver. The parent compound was also found in serum and kidney samples at 15 and 30 min, but it was not detected in urine where M6, M8, and M9 were the major metabolites, even at 24 h. *O*-demethyl AMB-CHMICA (M1) was the only compound detected in liver samples collected 48 h after injection.

### Biotransformation and pharmacokinetics

**Fig. 4:**
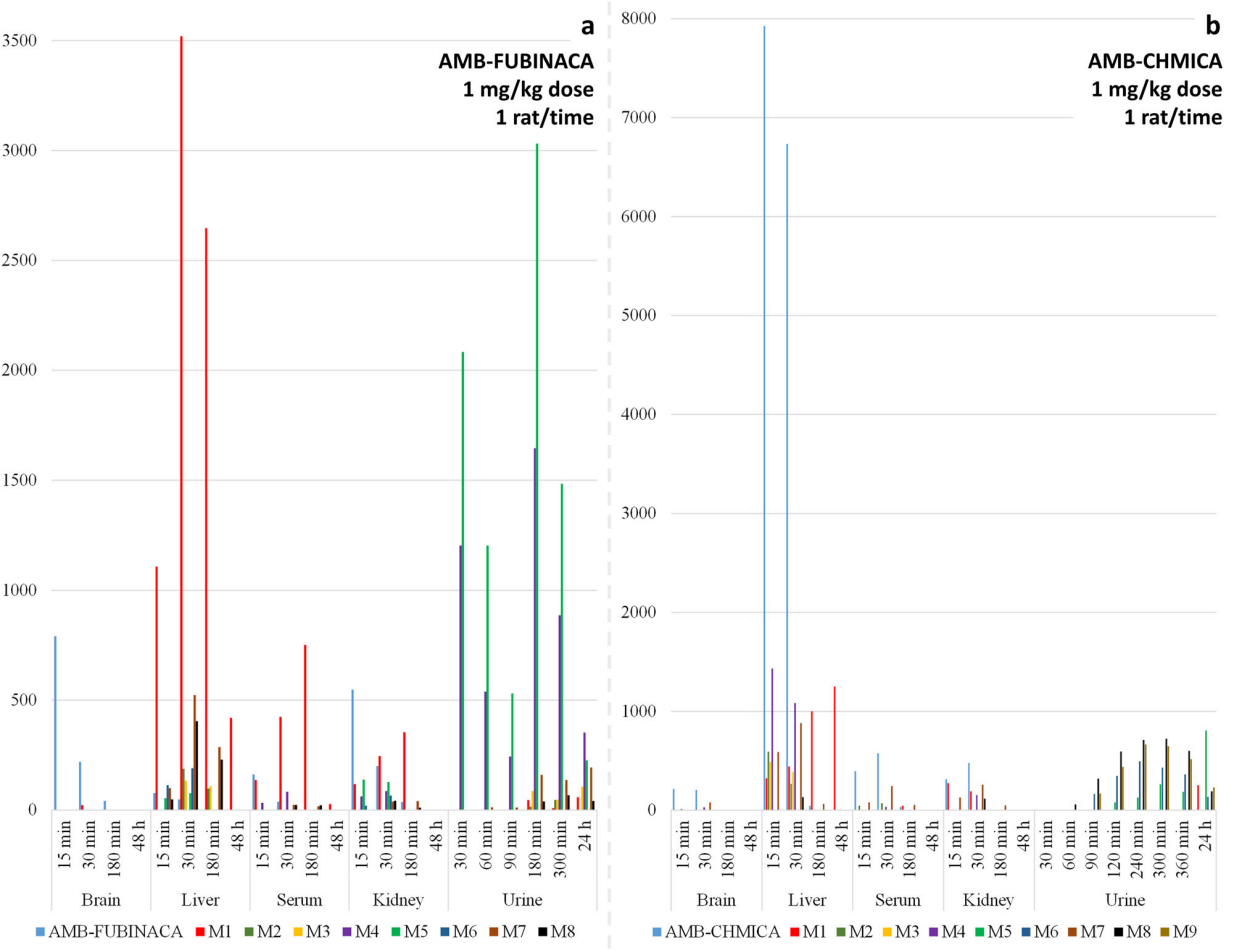
SCRAs and metabolites distribution over time. Distribution of the parent compound and metabolites of **a** AMB-FUBINACA and **b** AMB-CHMICA in the different matrices analysed over time. Stick height represents analytical response of the compound.

In spite of the structural similarities between both compounds, which share a methyl valinate moiety that is the main site of metabolism, relevant differences were observed in the proposed metabolic pathways in the studied SCRAs (Figs. 2 and 3). Interestingly, no phase II metabolites were detected. So, the intensities obtained for the phase I metabolites (Tables S1 and S2) were represented for each matrix at each sampling times in order to facilitate pharmacokinetics evaluation (Fig. 4). Additionally, the parent compound and *O*-demethyl metabolites were quantified in those samples in which they were detected (Table 3). It should be pointed out that a complete validation of the analytical method was not performed, as neither the extraction recoveries nor the accuracy and precision of the whole method were evaluated. Nevertheless, in order to increase confidence in the quantification, matrix effect was corrected by analysis of QCs samples and subsequent application of the corresponding correction factors. The obtained concentration values, measured in one sample, are therefore estimated concentrations. In most of the samples, signal suppression occurred, except for brain tissue, which produced signal enhancement for parent compounds. After matrix effects correction, analyte recoveries were between 60 and 80% for *O*-demethyl metabolites, and between 85 and 110% for the parent compounds.

**Table 3:** Quantification of parent compounds and *O*-demethyl metabolites in the different samples analysed.

Compound	Time	Brain (ng/g)	Liver (ng/g)	Kidney (ng/g)	Serum (ng/mL)	Urine (ng/mL)
AMB-FUBINACA	15 min	57.2	7.25	42.6	13.0	n.d.
	30 min	18.4	4.59	16.4	3.03	n.d.
	180 min	2.64	n.d.	3.36	n.d.	n.d.
	300 min	n.a.	n.a.	n.a.	n.a.	n.d.
	24 h	n.a.	n.a.	n.a.	n.a.	n.d.
	48 h	n.d.	n.d.	n.d.	n.d.	n.a.
*O*-demethyl AMB-FUBINACA	15 min	n.d.	111	12.5	24.9	n.d.
	30 min	3.83	356	27.4	76.8	n.d.
	180 min	n.d.	265	43.7	136	5.78
	300 min	n.a.	n.a.	n.a.	n.a.	1.13
	24 h	n.a.	n.a.	n.a.	n.a.	7.33
	48 h	n.d.	31.49	n.d.	5.72	n.a.
AMB-CHMICA	15 min	21.3	846	36.5	45.1	n.d.
	30 min	20.7	874	66.0	65.9	n.d.
	180 min	n.d.	4.64	n.d.	3.93	n.d.
	300 min	n.a.	n.a.	n.a.	n.a.	n.d.
	24 h	n.a.	n.a.	n.a.	n.a.	n.d.
	48 h	n.d.	n.d.	n.d.	n.d.	n.a.
*O*-demethyl AMB-CHMICA	15 min	n.d.	49.9	46.40	n.d.	n.d.
	30 min	n.d.	82.3	38.20	n.d.	n.d.
	180 min	n.d.	153	n.d.	10.2	n.d.
	300 min	n.a.	n.a.	n.a.	n.a.	n.d.
	24 h	n.a.	n.a.	n.a.	n.a.	37.7
	48 h	n.d.	218	n.d.	n.d.	n.a.

n.a.: Sample not available.

n.d.: Compound below the limit of detection.

In this work, *O*-demethylation reaction was observed, as described for these SCRAs in in vitro studies^19,27^, as well as for other SCRAs presenting the same moiety^29^. Nevertheless, AMB-FUBINACA was removed faster than AMB-CHMICA (Fig. 4 and Table 3). Fast hepatic metabolism of AMB-FUBINACA was already observed when using pooled human hepatocytes, in which after 2–3 min of incubation the *O*-demethyl AMB-FUBINACA was the major compound found in incubation media^19^. The *O*-demethyl AMB-FUBINACA metabolite was in fact the major compound detected in liver samples (111 ng/g at 15 min), while the concentration of the parent compound was 7 ng/g. On the contrary, AMB-CHMICA was found in liver at 846 ng/g, while its *O*-demethyl metabolite was present at lower concentration (50 ng/g). After 180 min of injection, AMB-FUBINACA was no longer detected in liver tissue and AMB-CHMICA was < 5 ng/g, while their *O*-demethyl metabolites were found at 265 and 154 ng/g, respectively. The concentration of *O*-demethyl AMB-FUBINACA decreased in liver after 180 min, whereas *O*-demethyl AMB-CHMICA was still increasing at this time (Table 3). The levels in liver for parents and metabolites matched with the concentrations found in serum, where AMB-FUBINACA was present at trace levels and descending over time while *O*-demethyl AMB-FUBINACA increased along the time, and AMB-CHMICA was detected at low sampling times while *O*-demethyl AMB-CHMICA was only found at 180 min (Table 3).

The concentration ratios *O*-demethyl metabolite/parent compound in the studied tissues illustrates the impact of the *O*-demethyl reaction, especially in liver and serum samples, as shown in Table 4. These results highlight that compounds with the same moiety, thus suffering the same biotransformations, can present important kinetic differences. Therefore, in vivo studies are needed for assessing the metabolism of NPS and to complement the information provided by in vitro approaches, which are useful to provide comprehensive information on the pharmacological behaviour on a certain tissue (for example, liver when using hepatocytes).

**Table 4:** *O*-demethyl metabolite/parent compound ratio in different samples analysed. The higher values, the higher concentration of *O*-demethyl metabolite.

Ratio	Time	Brain	Liver	Kidney	Serum
*O*-demethyl AMB-FUBINACA / AMB-FUBINACA	15 min	n.d.	15.4	0.29	1.91
	30 min	0.21	77.5	1.67	25.4
	180 min	n.d.	n.d.	13.0	n.d.
*O*-demethyl AMB-CHMICA / AMB-CHMICA	15 min	n.d.	0.06	1.27	n.d.
	30 min	n.d.	0.09	0.58	n.d.
	180 min	n.d.	33.1	n.d.	2.61

n.d.: Either metabolite or parent below the limit of detection.

Another common metabolic reaction observed for both compounds was the hydroxylation in the isopropyl group. After OH-isopropyl and *O*-demethyl reactions, AMB-FUBINACA and AMB-CHMICA presented differences in the subsequent reactions observed. In AMB-FUBINACA, the hydroxylated isopropyl was oxidised to carboxylic acid, observing two isomeric metabolites corresponding to the two carboxylable methyl groups of the valinate moiety (Fig. 2). In the case of AMB-CHMICA, it was metabolised to lactone (or alkyl insaturation) without observing carboxylation (Fig. 3).

Regarding the other important moiety of SCRAs, the indole/indazole *N*-functionalization, relevant differences were also found. AMB-FUBINACA presented an *N*-dealkylation followed by a *N*-methylation (Fig. 2), which is in agreement with the results obtained in human hepatocytes incubation^19^. However, this was not observed in this study for AMB-CHMICA but when using hepatocyte incubation^27^. The metabolic pathway of AMB-CHMICA goes through hydroxylation in the cyclohexylmethyl, produced directly in the parent compound, after *O*-demethyl, after OH-isopropyl, or after lactonization (Fig. 3). Indeed, the *N*-dealkyl and *N*-dealkyl-*N*-methyl AMB-FUBINACA metabolites, and lactone+OH-cyclohexylmethyl AMB-CHMICA metabolites, were the major compounds observed in urine samples. This is not surprising, as these metabolites are the most polar ones, as it will be discussed in the following section.

It can be concluded that the metabolic pathways for AMB-FUBINACA and AMB-CHMICA based on Sprague-Dawley rats in vivo experiments present important differences despite they have similar chemical structures. This is contrary to the information provided by hepatic in vitro studies that suggested similar metabolic reactions. In this sense, it should be taken into account the differences between rats and humans regarding the isoform composition, expression and catalytic activities of drug-metabolising enzymes^35^. For example, although CYP2E1 does not show large interspecies differences, this does not occur for other CYP isoforms, like CYP1A, −2C, −2D, and −3A, which display interspecies differences in terms of catalytic activity^35^. Therefore, some caution should be taken before extrapolating metabolism data from animal models onto humans.

### Tissue distribution and barrier permeability

The distribution of the parent compounds and the detected metabolites through the studied tissues over time can also be assessed from the information shown in Fig. 4 and Table 3.

As previously commented, parent compound and *O*-demethyl metabolite were found in liver and serum samples for both SCRAs. In the case of AMB-CHMICA, (Fig. 4b and Table 3) as the parent concentration decreases over time, the *O*-demethyl metabolite increases, as well as other metabolites, such OH-isopropyl (M4) and lactone (M7), at low sampling times. Nevertheless, parent compound was also detected in serum, brain and kidney at 15 and 30 min, being the major compound at these sampling times.

Similarly, AMB-FUBINACA was also detected at 15 and 30 min in serum, brain, liver and kidney. The fast *O*-demethyl reaction promotes *O*-demethyl AMB-FUBINACA to be the major compound in liver at the three sampling times (Fig. 4a and Table 3), indicating that the *O*-demethylation predominately originates from hepatic metabolism, being catalysed by different enzymes^36,37^. The pharmacokinetics of this SCRA and its major metabolite in different tissues was the following: the concentration of AMB-FUBINACA decreased along the time in serum, liver and kidney, while the *O*-demethyl metabolite increased. OH-isopropyl (M6) and carboxylated metabolites (M7 and M8) were also found in liver samples at 30 and 180 min, presenting high analytical responses. In the case of kidney, and prior to excretion, *N*-dealkyl (M4) and *N*-dealkyl-*N*-methyl (M5) metabolites were important compounds in terms of analytical response.

The differences in the parent compound and metabolites tissue distribution may be related with the polarity of the compounds. The high concentrations of AMB-CHMICA and low relative amount of *O*-demethyl metabolite, compared to AMB-FUBINACA, (Table 3) might be due to the lower polarity of AMB-CHMICA, whose higher lipophilic properties would lead to a higher bioaccumulation in liver. In fact, this compound presented higher chromatographic retention time in reversed-phase liquid chromatography, which reveals its lower polarity in comparison with AMB-FUBINACA.

The ratio between the liver tissue concentrations and serum concentration allowed to assess the distribution between liver and serum and to determine the bioaccumulation in this organ. (Tables 3 and 5). This ratio liver/serum for AMB-CHMICA was higher than for AMB-FUBINACA at all the sampling times, indicating that AMB-CHMICA is highly bioaccumulated in liver. A similar behaviour was observed for the main metabolites, as *O*-demethyl AMB-FUBINACA presented higher concentration in serum (25 ng/mL at 15 min) than *O*-demethyl AMB-CHMICA (10 ng/mL at 180 min) while the concentrations of both metabolites were rather similar in liver (110 and 150 ng/g, respectively). This difference suggests that *O*-demethyl AMB-FUBINACA is secreted from hepatocytes to the blood stream easier than *O*-demethyl AMB-CHMICA and thus, is less bioaccumulated. As both compounds have a methyl valinate moiety, the polarity difference comes from the indazole/indole *N*-functionalisation: fluorophenylmethyl in AMB-FUBINACA, and cyclohexylmethyl in AMB-CHMICA.

**Table 5:** Tissue (ng/g)/serum (ng/mL) ratio for parent compound and *O*-demethyl metabolites.

Compound	Time	Brain/serum	Liver/serum	Kidney/serum
AMB-FUBINACA	15 min	4.38	0.56	3.27
	30 min	6.05	1.52	5.42
	180 min	n.d.	n.d.	n.d.
*O*-demethyl AMB-FUBINACA	15 min	n.d.	4.47	0.50
	30 min	0.05	4.63	0.36
	180 min	n.d.	1.94	0.32
AMB-CHMICA	15 min	0.47	18.8	0.81
	30 min	0.31	13.3	1.00
	180 min	n.d.	1.18	n.d.
*O*-demethyl AMB-CHMICA	15 min	n.d.	n.d.	n.d.
	30 min	n.d.	n.d.	n.d.
	180 min	n.d.	15.0	n.d.

n.d.: Compound below the limit of detection in tissue and/or serum.

The SCRAs potency and efficiency are related to their affinity to the CB1 and CB2 cannabinoid receptors, present in the central and peripheral nervous system^5,38^. In fact, CB1 receptors are also present in brain^39^ and thus, the potency of a certain SCRA can be also affected by the permeability of this compound through the blood-brain barrier. A recent study illustrated that the different moieties of synthetic cathinones play a pivotal role on their permeability through the blood-brain barrier^40^. Therefore, it is important to assess the presence of these SCRAs and metabolites in the brain tissue samples. As shown in Table 3, AMB-FUBINACA and AMB-CHMICA were found in brain samples at low sampling times, with the highest concentration at 15 min (57 and 21 ng/g, respectively). Nevertheless, if the ratio brain/serum is calculated, AMB-FUBINACA presents higher permeability than AMB-CHMICA (up to ten times) at measured time points, as it can be observed in Table 5.

All these results indicate that compounds with similar metabolic pathways can suffer important differences in their distribution on different tissues, as well as in permeability through, for example, the blood-brain barrier.

### Excretion and biomarker proposal

The last step after elucidating the SCRAs metabolites and determining their distribution in tissues and pharmacokinetics was the excretion assessment. It was expected finding in urine the most polar metabolites, which actually occurred in this work, as shown Fig. 4. For both compounds, those metabolites with lower chromatographic retention time (Tables 1 and 2) were the major compounds in urine samples.

The two major urinary AMB-FUBINACA metabolites were the *N*-dealkyl (M4) and *N*-dealkyl-*N*-methyl (M5), found in all the urine samples collected, even 24 h after injection. These metabolites are the first compounds detected in the samples collected at 30, 60, and 180 min, while traces of additional metabolites were also observed at higher times, including the major metabolite (*O*-demethyl AMB-FUBINACA) detected in the 24 h urine sample (Fig. 4a). In the case of AMB-CHMICA, the urinary metabolites with higher analytical response were the lactone+OH-cyclohexylmethyl (M8 and M9) and the *O*-demethyl+OH-cyclohexylmethyl (M5 and M6), as shown in Fig. 4b. These compounds were detected at 90 min after injection, with the maximum concentration found between 240 and 300 min after injection. These four metabolites, together with *O*-demethyl AMB-CHMICA, were also detected in the 24 h urine sample.

Due to the non-polarity of SCRAs, which are retained and mostly metabolised in liver, it is uncommon to find the intact molecule in urine. In spite of the methyl valinate metabolism, additional metabolic reactions are needed in the indazole/indole *N*-functionalisation moiety to allow the excretion of these compounds. The metabolism of AMB-FUBINACA goes through *N*-demethylation and posterior *N*-methylation (Fig. 2), while for AMB-CHMICA, OH-cyclohexylmethyl is observed (Fig. 3). In both cases, the urinary metabolites suffer biotransformations in the methyl valinate moiety: *O*-demethyl for AMB-FUBINACA (Fig. 2), and *O*-demethyl and lactone formation for AMB-CHMICA (Fig. 3). Surprisingly, no phase II metabolites were found in urine samples for any of the SCRAs studied, even though they were directly searched by expected biotransformation, and also indirectly by a common fragmentation pathway.

One of the most important outputs of metabolism and pharmacokinetics experiments is the proposal of the most suitable biomarkers of consumption, classified as short-term or long-term metabolites, depending on their prevalence over time. The *O*-demethyl metabolite of AMB-FUBINACA was found to be the most adequate biomarker in liver, serum, and kidney (Fig. 4a). Indeed, *O*-demethyl AMB-FUBINACA has been used to confirm the consumption of this NPS in toxicological analysis, and it was detected in blood samples^41^. In our study, this metabolite was also detected in liver and serum samples 48 h after injection at 31 ng/g and 6 ng/mL, respectively, therefore being an excellent post-mortem biomarker (Fig. S20a). Nevertheless, *O*-demethyl AMB-FUBINACA can also be a consumption biomarker of AB-FUBINACA, as reported in literature^42,43^. Therefore, additional biomarkers should be monitored for AMB-FUBINACA in order to discriminate both SCRAs consumption. Additional post-mortem biomarkers in liver samples can be M7 and M8 (isopropyl carboxylic acid), as they were also detected at 180 min after injection (Fig. 4a). In a massive intoxication with AMB-FUBINACA in 2016, *O*-demethyl AMB-FUBINACA was found in serum and urine from different patients, but no additional metabolites were searched for^44^. We propose to use *O*-demethyl AMB-FUBINACA as biomarker in serum and liver, and *N*-dealkyl AMB-FUBINACA (M4) and *N*-dealkyl-*N*-methyl AMB-FUBINACA (M5) as short-term biomarkers in urine. As long-term metabolites, it would be preferably to include also M7 (Fig. 4a). The M4 and M5 metabolites were also found using human hepatocytes incubations^19^, and therefore, it can be expected to detect both compounds in authentic urine samples from human AMB-FUBINACA users.

Regarding AMB-CHMICA, different compounds could be used as biomarkers too, depending on the matrix and time after consumption. In the case of liver samples for post-mortem analysis, the parent compound seems to be the most adequate short-term biomarker, together with OH-isopropyl (M4) and lactone (M7) as they were detected with relatively high analytical response in this tissue (Fig. 4b). Additionally, *O*-demethyl AMB-CHMICA should be considered as long-term metabolite in liver. This metabolite was found at 218 ng/g in the sample collected 48 h after injection (Fig. S20b), illustrating its high bioaccumulation in liver and thus, its suitability to be used as post-mortem biomarker. Moreover, the *O*-demethyl+OH-cyclohexylmethyl (M6) isomer, and the two isomers lactone+OH-cyclohexylmethyl (M8 and M9) presented the highest analytical responses in urine samples collected between 90 and 360 min (Fig. 4b). Nevertheless, the major compounds detected in the 24 h urine were *O*-demethyl AMB-CHMICA (38 ng/mL) and the *O*-demethyl+OH-cyclohexylmethyl isomer (M5) (Fig. 4b). *O*-demethyl AMB-CHMICA and M6 have also been reported in human hepatocytes incubations^27^, and, therefore, these compounds are expected in authentic human samples to confirm (post-mortem) the consumption by the analysis of liver and urine samples, respectively. In the case of M6, M8, and M9, these are the major compounds detected in Sprague-Dawley rat urine, and should also be included in the toxicological analysis in spite of the no evidences of the formation of these metabolites in hepatocyte incubations.

As a final note, in this work, an in-depth evaluation of two structurally-related SCRAs, AMB-FUBINACA and AMB-CHMICA, has been made. These compounds were selected as they share a methyl valinate moiety, which is the main metabolic site according to data reported from in vitro experiments. The aim was to evaluate the differences between both compounds in terms of metabolism, pharmacokinetics, tissue distribution, bioaccumulation, and urinary excretion in male Sprague-Dawley rats by the analysis of brain, liver, kidney, serum, and urine samples collected at different times. Identification/elucidation of metabolites, as well as quantification of parent compounds and O-demethyl metabolites, was performed by UHPLC-HRMS, and potential metabolites were searched and elucidated based on the observed accurate-mass fragmentation. For AMB-FUBINACA, 8 phase I metabolites were detected, while 9 phase I metabolites were elucidated for AMB-CHMICA. All the metabolites were produced by hydroxylation, oxidation, and dealkylation reactions. Our results demonstrated important metabolic differences for both compounds, as well as different velocities in the main metabolic reaction (*O*-demethylation). While AMB-FUBINACA was rapidly metabolised through this biotransformation, AMB-CHMICA was highly bioaccumulated in liver and demethylation took more time. Regarding brain tissue samples, the permeability was evaluated based on the ratio of the concentrations found in brain and in serum at a certain time, showing that AMB-FUBINACA was around ten-fold more permeable than AMB-CHMICA. The excretory metabolites were also assessed, confirming the prevalence of the most polar metabolites in urine. Based on the obtained results, the most suitable biomarkers of consumption were proposed. For the determination of AMB-FUBINACA in intoxication and post-mortem analysis, *O*-demethyl metabolite was appropriate as short-term and long-term biomarker for liver and serum analysis, and it was detected 24 h after injection. Moreover, indazole *N*-dealkyl and *N*-dealkyl-*N*-methyl metabolites were found the most adequate for short-term urine samples, as well as for long-term samples together with the *O*-demethyl and isopropyl carboxylic acid metabolite. For AMB-CHMICA, the parent compound was found an appropriate short-term biomarker in liver and urine, while *O*-demethyl is an excellent long-term metabolite for liver analysis. The metabolites *O*-demethyl+OH-cyclohexyl and lactone+OH-cyclohexyl were found in urine at 24 h after injection, as well as in short-term urine samples.

This study shows that SCRAs are complex compounds from a pharmacological point of view, as well as the useful and wide information that can be gathered from in vivo experiments complemented by a detailed analytical work by using HRMS.

## Methods

### Reagents and chemicals

AMB-FUBINACA research chemical was provided by Energy Control (ABD Foundation, Barcelona, Spain). Its characterisation and purity were assessed by UHPLC-HRMS and nuclear magnetic resonance as described in the literature^45,46^. AMB-CHMICA, *O*-demethyl AMB-FUBINACA (purchased as AB-FUBINACA carboxyl acid metabolite), and *O*-demethyl AMB-CHMICA (MMB-CHMICA *O*-demethyl acid metabolite) analytical reference standards were obtained from Chiron AS (Trondheim, Norway). For UHPLC-HRMS analysis, ultrapure water was obtained by purifying demineralised water using a Milli-Q system from Millipore (Bedford, MA, USA). LC-MS grade acetonitrile (ACN), LC-MS grade formic acid, sodium hydroxide (NaOH), and ethanol were purchased from Scharlau (Scharlab, Barcelona, Spain). Leucine enkephalin acetate salt hydrated (>95%) was purchased from Merck (Darmstadt, Germany). Physiological saline solution was acquired from Laboratorios ERN (Barcelona, Spain).

### Animal experiments

8 male Sprague-Dawley rats (8 weeks old, weighing between 385 and 425 g) were used as animal models (Janvier Labs, Le Genest-Saint-Isle, France). Animals were housed individually in polypropylene plastic cages under controlled temperature (24 ± 2 °C) and lighting conditions (12 h:12 h; lights ON at 8 AM), with *ad libitum* access to food and water. Before SCRA injection, animals were handled and habituated to the experimental room for 1 week. All the experiments were approved by the Committee of Ethics and Animal Experimentation of Universitat Jaume I (2019/VSC/PEA/048) and treated throughout according to the European Union Council Directive of June 3rd, 2010 (6106/1/10 REV1).

SCRAs were dosed intraperitoneally at 1 mg/kg. This dose was selected based on similar studies reported in literature^47^. The solution used for injection was prepared at 1 mg/mL in physiological saline solution containing 5% ethanol. Between 385 and 425 µL of that solution were injected depending on animal weight. Blood (serum), liver, brain, and kidney samples were dissected 15, 30, and 180 min after injection (previously anesthetised with CO_2_ and decapitated immediately). Blood was collected mainly from carotid arteries and jugular veins and kept at room temperature for 30 min to elicit coagulation. Then, blood was centrifuged 10 min at 2000 rcf at 4 °C in order to separate serum from sedimented clot. Liver, kidney, and brain were rapidly dissected and snap-freezing in liquid nitrogen. One animal was used for each dissection time. Additional serum, liver, brain, and kidney samples were obtained from an animal 48 h after being SCRA-dosed, and used as control. Urine samples were collected at 30, 60, 90, 120, 180, 240, 300, and 360 min, together with an additional sample at 24 h (composite urine sample between 360 min and 24 h, collected from animal dissected at 48 h). Urine samples were obtained from the bottom of the cage at the indicated time. Additionally, urine sample collected from one of the animals prior to injection was used as control. Samples were snap-frozen in liquid nitrogen after collection, and preserved at −23 °C until analysis.

When the liver (and also brain and kidney) tissue was homogenised, hepatocytes, capillaries leading content to the hepatocytes for clearance and from the hepatocytes after secretion, as well as the biliary canaliculi were mixed. Therefore, the liver tissue represented the whole organ, without differentiating any compartment.

### Sample treatment

Solid samples (brain, liver, and kidney) were crushed with dry ice using an electric grinder. 0.1 g were accurately weighed (±0.1 mg) in 1.5 mL polypropylene tubes, and 0.3 mL of acetonitrile acidified with 1% of formic acid were added for extraction. After that, samples were sonicated for 5 min, followed by 30 min of vortex agitation. Samples were then frozen for 30 min in order to increase protein removal. Finally, they were centrifuged at 12,500 rfc during 15 min, and the supernatant was 10-fold diluted with ultrapure water for UHPLC-HRMS analysis.

In the case of liquid samples (serum and urine), 0.1 mL were diluted with 0.3 mL of acetonitrile acidified with 1% of formic acid in 1.5 mL polypropylene tubes, followed by a 1 min vortex agitation in order to produce protein precipitation, as rat urine also produced a visible protein precipitate. Similarly to solid samples, samples were frozen for 30 min, centrifuged at 12,500 rfc for 15 min, and the supernatant 10-fold diluted with ultrapure water for UHPLC-HRMS analysis.

For parent compounds and *O*-demethyl metabolites estimated quantification, a calibration line in solvent from 2.5 to 250 ng/mL was included in each batch analysis, as well as different quality control samples (QCs) prepared with blank extracts and spiked at 200 ng/mL for evaluating matrix effect and apply a correction factor.

### Instrumentation

Samples were analysed using an Acquity ultra-performance liquid chromatography (UPLC) system (Waters Corp, Mildford, MA, USA) coupled to a Xevo G2 QTOF (Waters Corp, Manchester, UK) mass spectrometer with quadrupole time-of-flight (QTOF) hybrid mass analyser.

Chromatographic separation was performed using a Cortecs T3 100 ×2.1 mm, 1.6 µm particle size analytical column (Waters Corp, Wexford, Ireland), maintained at 40 °C. Mobile phases were water (A) and ACN (B), both with 0.01% formic acid, delivered at a flow rate of 0.3 mL/min as follows: 5% of B at 0 and 0.5 min, 99% of B at 12 min linearly increased, 99% of B at 14.5 min, 5% of B at 14.6 min and maintained until 16 min. Injection volume was 50 µL.

HRMS system was equipped with an orthogonal Z-spray electrospray (ESI) source operating in positive and negative ionisation mode, working at 1.0 and −1.5 kV, respectively, and a cone voltage of 20 V. Nitrogen was used as desolvation and nebulising gas (1000 L/h), as well as cone gas (80 L/h), while Argon 99.995% (Nippon Gases, Valencia, Spain) was used as collision gas. Source temperature was established at 120 °C and desolvation temperature at 600 °C. The TOF resolution was ∼20,000 at FWHM in positive (*m/z* 556) and negative (*m/z* 554) ionisation modes, acquiring data from *m/z* 50 to 1000 using MS^E^ acquisition mode. Briefly, two functions were sequentially acquired: a low energy function (LE), for obtaining information about (de)protonated molecule and adducts (if exist), and a high energy function (HE), applying a collision energy ramp from 15 to 40 eV, for obtaining information about fragmentation. Mass-axis was daily calibrated from *m/z* 50 to 1000 using a 1:1 mixture of 0.05 M NaOH:5% formic acid, diluted 1:25 with ACN:water (80:20). For accurate-mass measurements, 2 µg/mL of leucine enkephalin solution in ACN:water (50:50) with 0.1% formic acid was used as lock-mass, pumped at a flow rate of 20 µL/min, using (de)protonated molecule to recalibrate the mass axis. MS data were acquired and processed using MassLynx data station operation software version 4.1 (Waters).

### Metabolite detection strategy

The analytical strategy used for detection and elucidation of metabolites of both SCRAs comprised three steps: suspect screening of reported metabolites, searching for expected biotransformations, and application of the common fragmentation pathway strategy.

First, the in vitro phase I metabolites reported in literature for AMB-FUBINACA^19^ and AMB-CHMICA^27^ were searched for in samples following a target and suspect screening strategy using ChromaLynx XS application (MassLynx v4.1, Waters Corp.). Information about chromatographic retention time and fragmentation for parent compounds and *O*-demethyl metabolites was used for the target identifications, while fragment ions reported for metabolites in the literature were included for suspect detections and identifications. The software automatically performed EICs to the *m/z* corresponding to the reported metabolites, with an extraction window of ±5 mDa, for (de)protonated molecules (searched in the LE function) and fragment ions (HE function). EICs were also extracted in control matrix samples in order to discard false positives. Compounds with at least one reported accurate-mass fragment ion were considered as identified metabolite in this step.

In a second step, phase I and phase II metabolites were searched based on expected biotransformations, using the MetaboLynx XS application (MassLynx v4.1, Waters Corp.). A list of potential reactions (and combinations) that could occur based on SCRAs structures was used for obtaining putative metabolites. The EICs (±5 mDa extraction window) corresponding to these potential metabolites were automatically performed in control and “drug” samples (LE function), and those peaks only present in rats injected with SCRAs, with a signal difference of ×10, were further investigated. Further information about expected biotransformation for metabolite detection can be found in literature^48,49^.

**Fig. 5:**
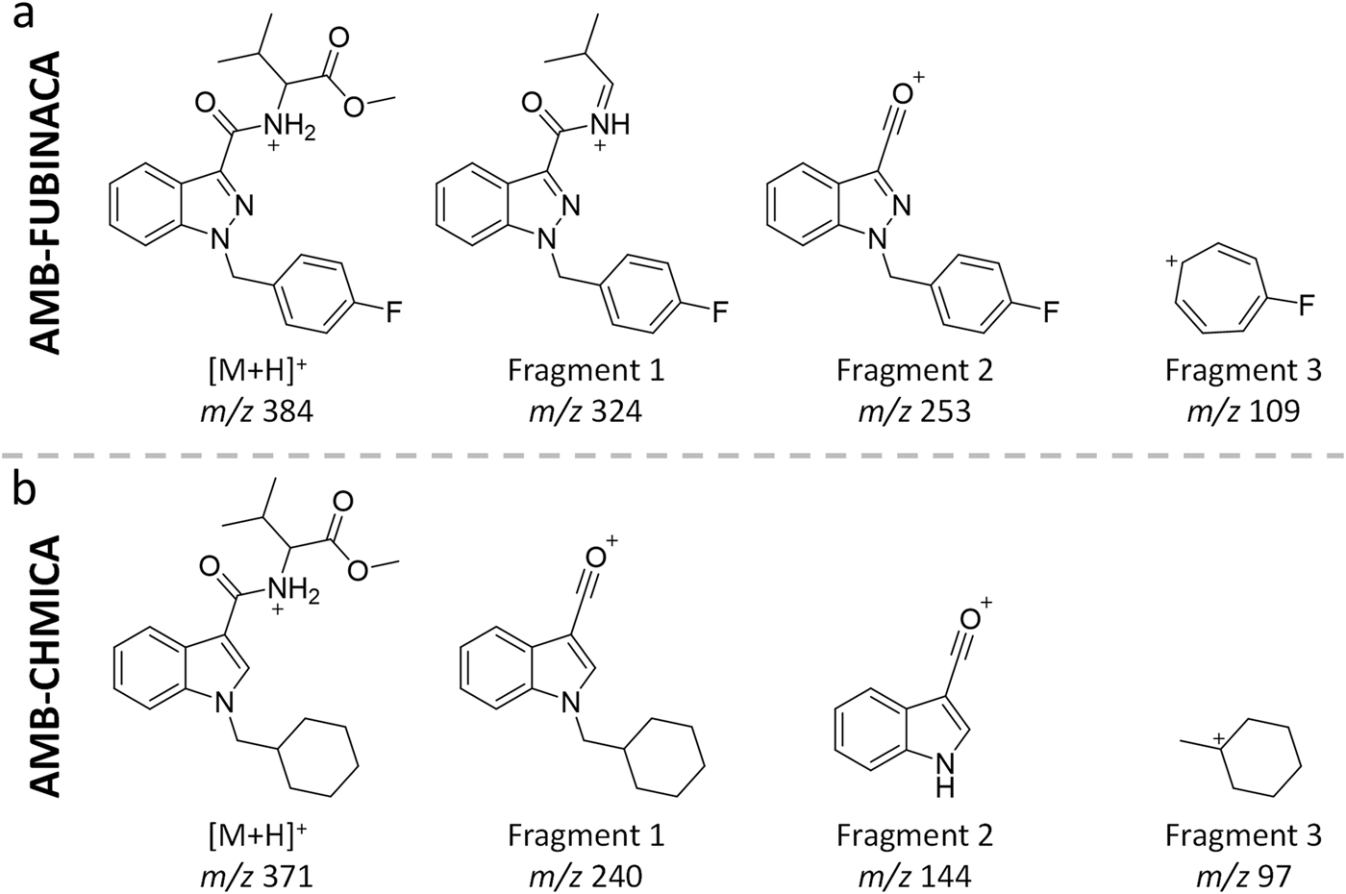
Proposed fragmentation for the two SCRAs. Proposed chemical structure for the accurate-mass fragments observed for **a** AMB-FUBINACA and **b** AMB-CHMICA. Below the proposed structure of each fragment ion it is displayed its corresponding *m/z*.

The last step consisted of an additional metabolite searching based on common fragmentation pathways with the parent compound and with metabolites detected in the previous steps. This strategy is based on the premise that metabolites share some fragment ions with their parent compound, corresponding to unaltered moieties^50^. In the case of SCRAs, they present similar fragmentation behaviour based on heteroatom bond disconnection, as shown in Fig. 5 and as reported in literature^19^. To this aim, EIC (±5 mDa extraction window) of fragment ions observed were obtained in the HE function, in order to detect additional peaks not observed in the suspect screening and expected biotransformation approach. The searching of additional metabolites by the common fragmentation pathway was complemented following the described strategy to the fragment ions from potential metabolites.

### Reporting summary

Further information on research design is available in the Nature Research Reporting Summary linked to this article.

## Supplementary information

Supplemental Material

Reporting Summary

## Data Availability

Authors can confirm that all relevant data are included in the article and/or its supplementary information files.
