# Supplementary material for: In-depth comparison of the metabolic and pharmacokinetic behaviour of the structurally related synthetic cannabinoids AMB-FUBINACA and AMB-CHMICA in rats
Source: Commun Biol. 2022 Feb 24;5:161. doi: 10.1038/s42003-022-03113-5 (PMC8873228; doi:10.1038/s42003-022-03113-5)
Supplement: Supplementary file 1 — Supplemental Material [file 42003_2022_3113_MOESM1_ESM.pdf]

Supplementary Information

In-depth comparison of the metabolic and pharmacokinetic behaviour of the structurally related synthetic cannabinoids AMB-FUBINACA and AMB-CHMICA in rats.

David Fabregat-Safont[1], María Mata-Pesquera[1], Manuela Barneo-Muñoz[2], Ferran Martinez-Garcia[2], Marie Mardal[3], Anders B. Davidsen[3], Juan V. Sancho[1], Félix Hernández[1], María Ibáñez[1].

[1] Environmental and Public Health Analytical Chemistry, Research Institute for Pesticides and Water (IUPA), University Jaume I, Avda. Sos Baynat s/n, 12071, Castellón, Spain.

[2] Predepartmental Unit of Medicine, Unitat Mixta de Neuroanatomia Funcional NeuroFun-UVEG-UJI, University Jaume I, Castellón, Spain.

[3] Section of Forensic Chemistry, Department of Forensic Medicine, University of Copenhagen, Copenhagen, Denmark.

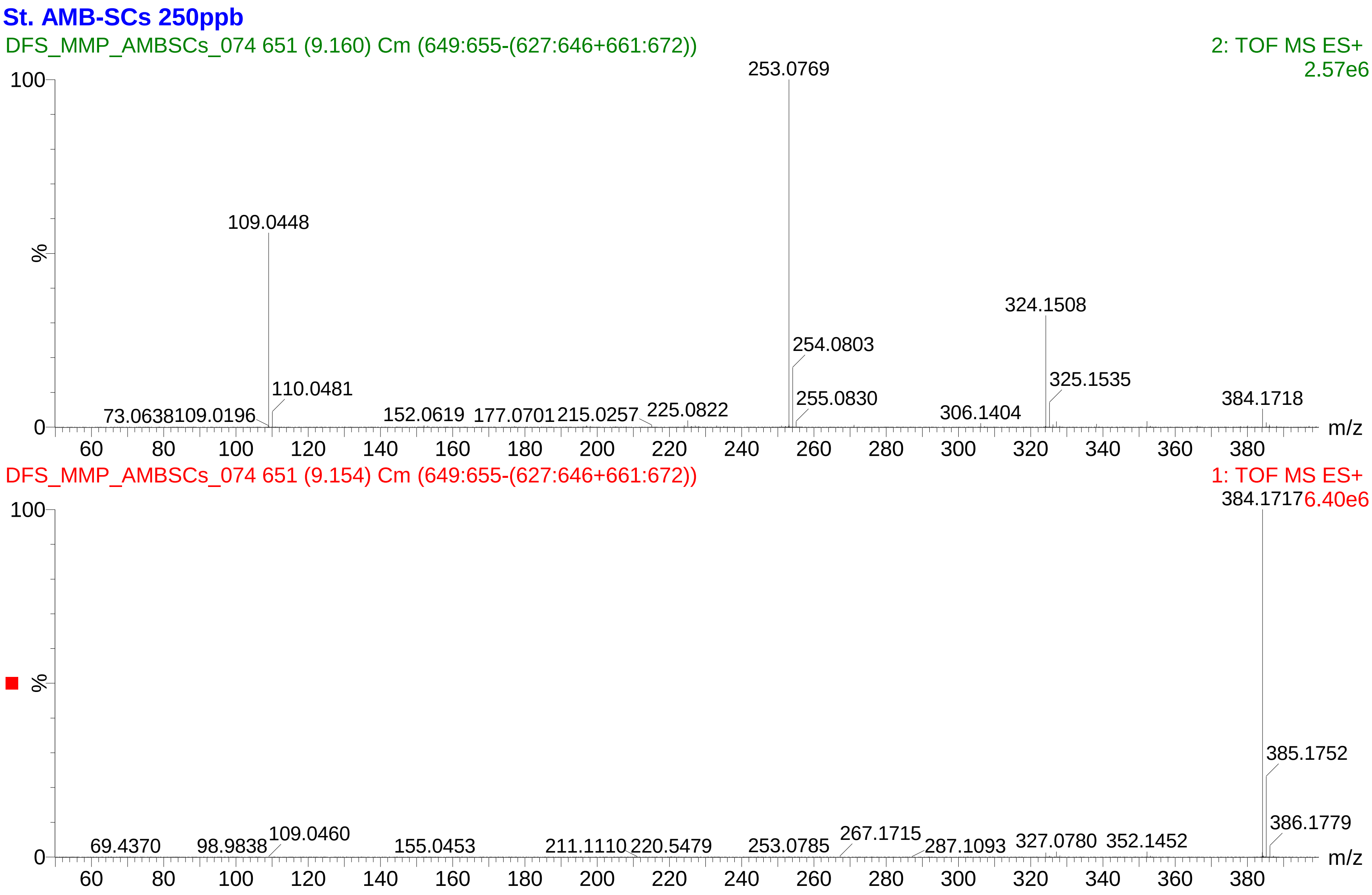

Fig. S1. Low Energy (Function 1) and High Energy (Function 2) spectra for AMB-FUBINACA.

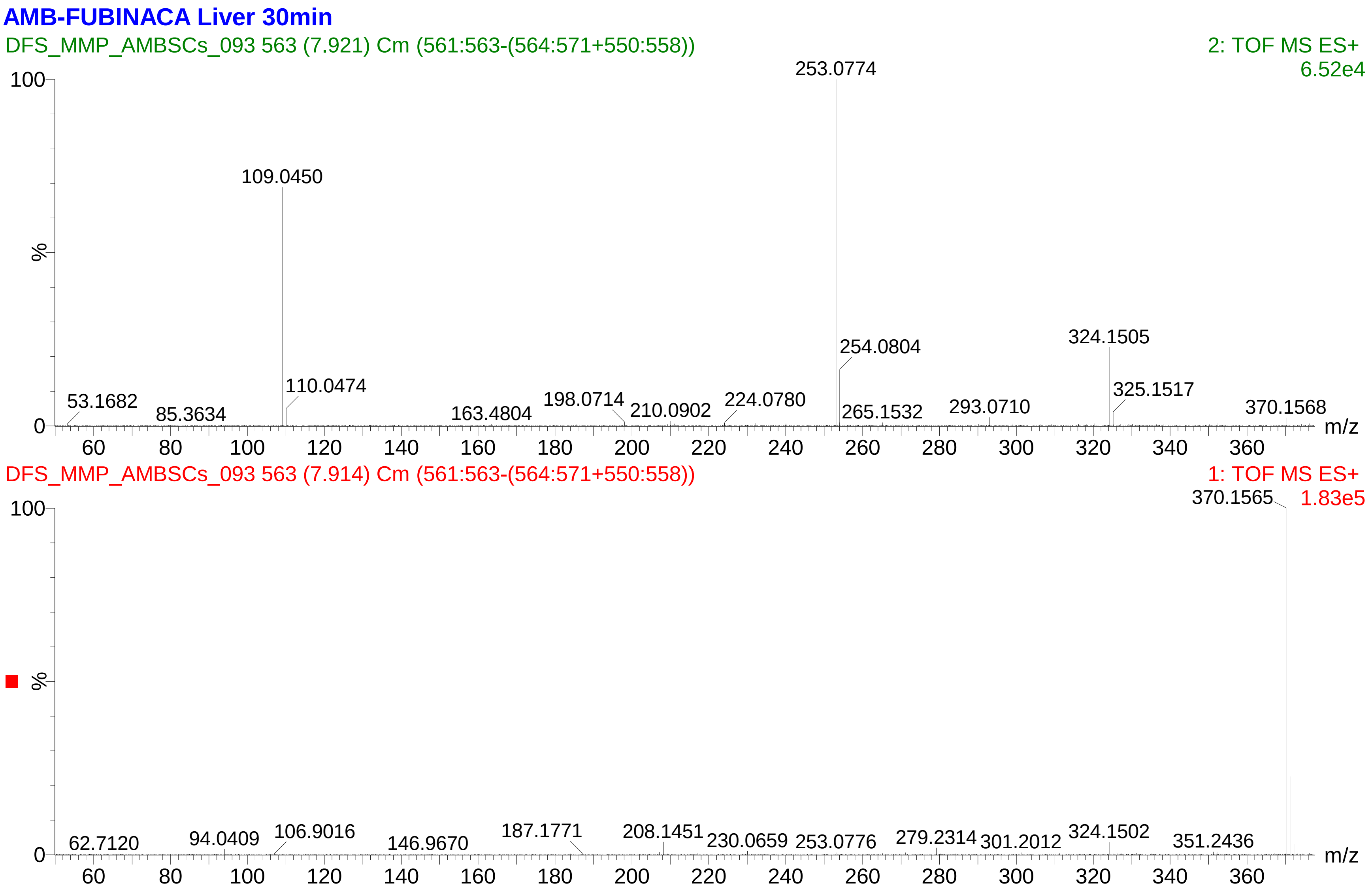

Fig. S2. Low Energy (Function 1) and High Energy (Function 2) spectra for AMB-FUBINACA M1.

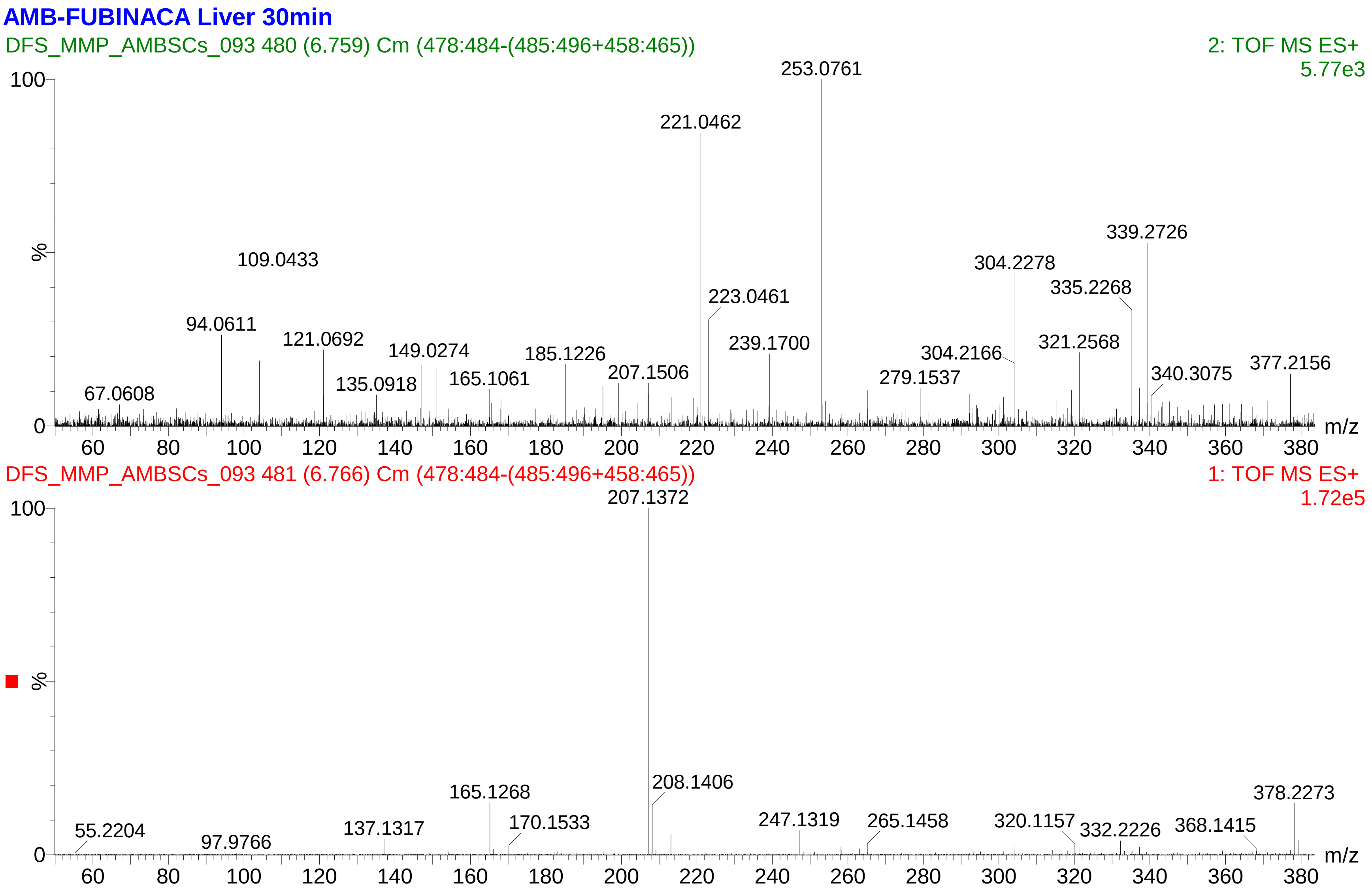

Fig. S3. Low Energy (Function 1) and High Energy (Function 2) spectra for AMB-FUBINACA M2.

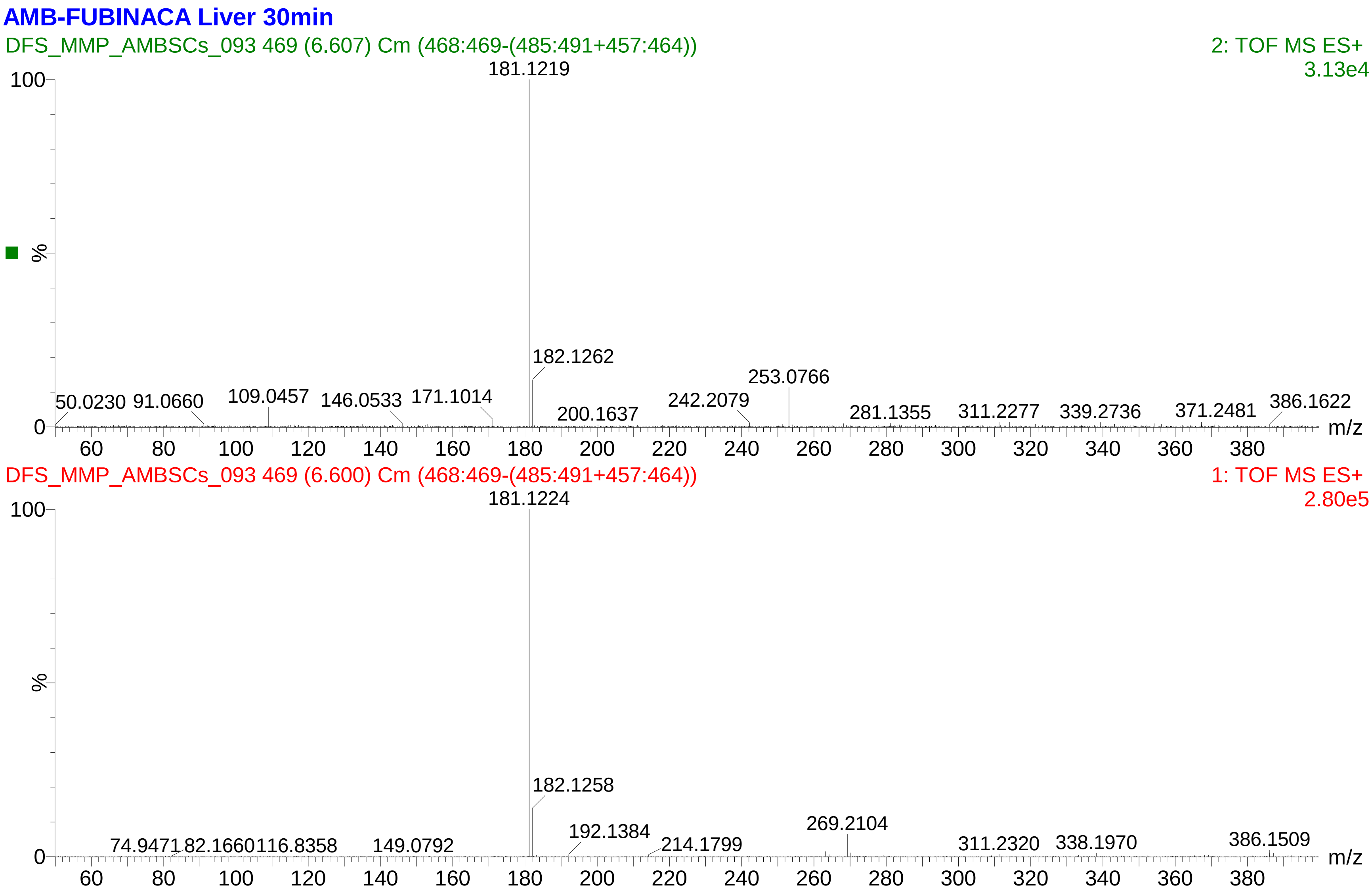

Fig. S4. Low Energy (Function 1) and High Energy (Function 2) spectra for AMB-FUBINACA M3.

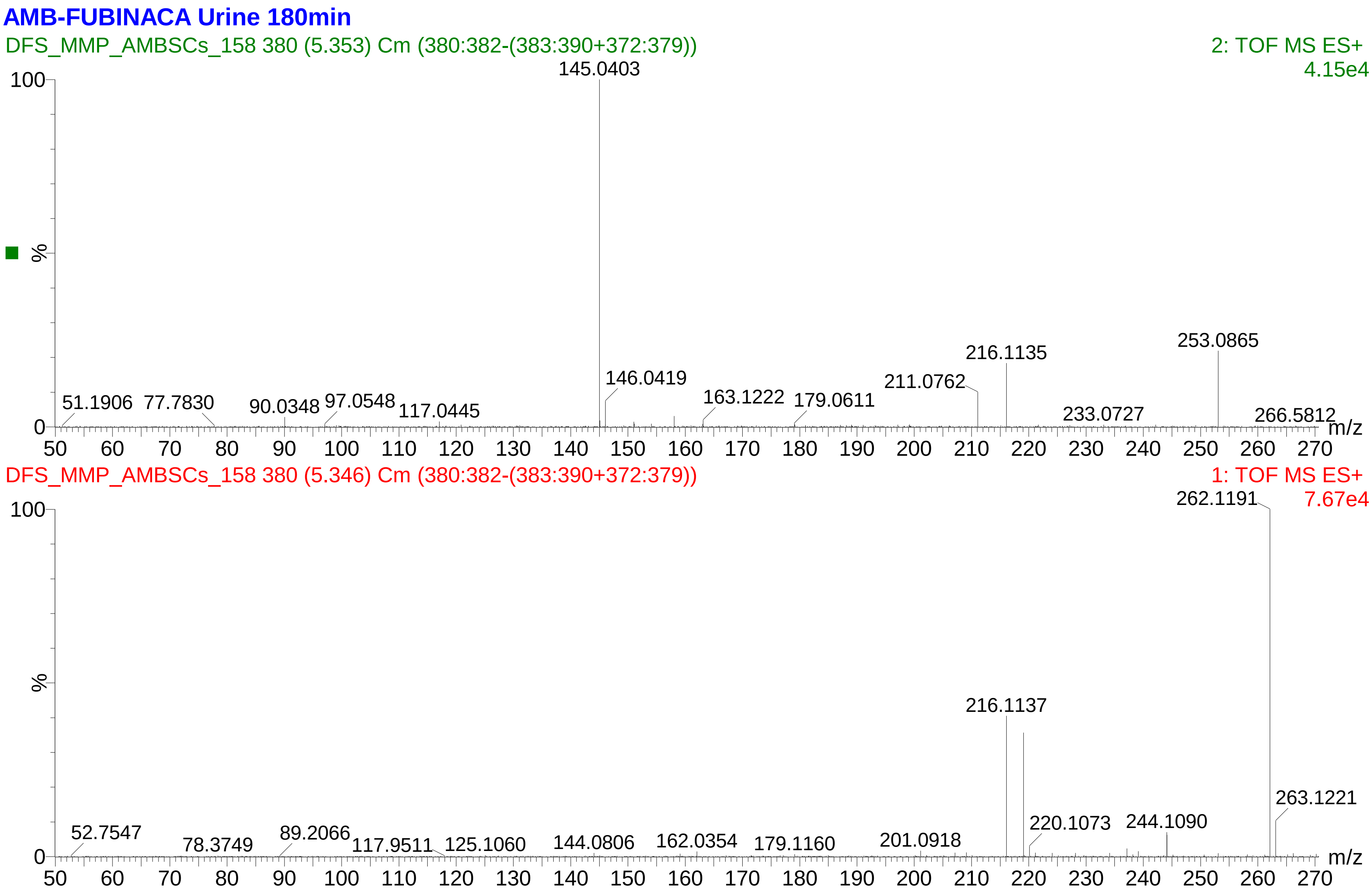

Fig. S5. Low Energy (Function 1) and High Energy (Function 2) spectra for AMB-FUBINACA M4.

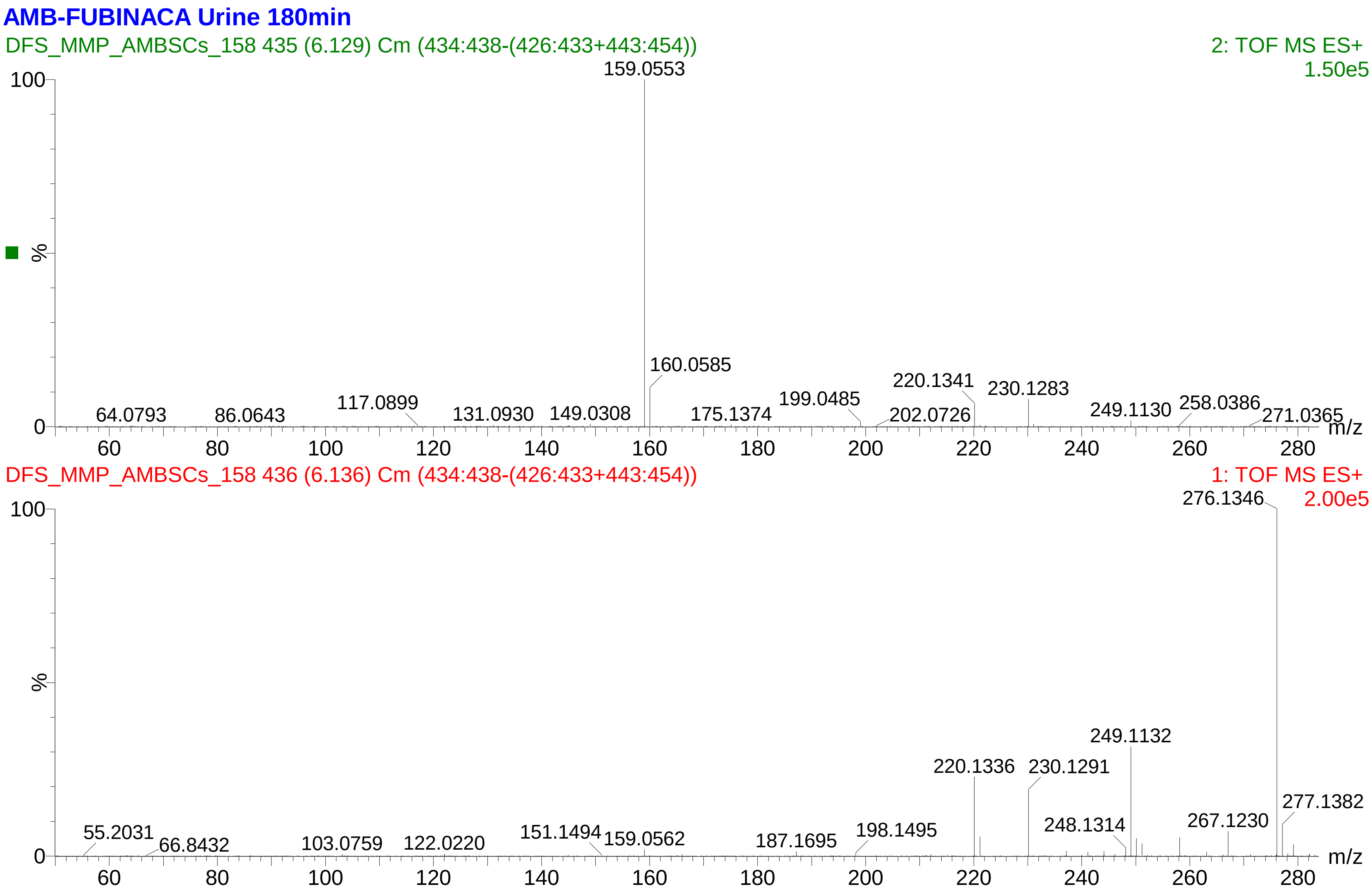

Fig. S6. Low Energy (Function 1) and High Energy (Function 2) spectra for AMB-FUBINACA M5.

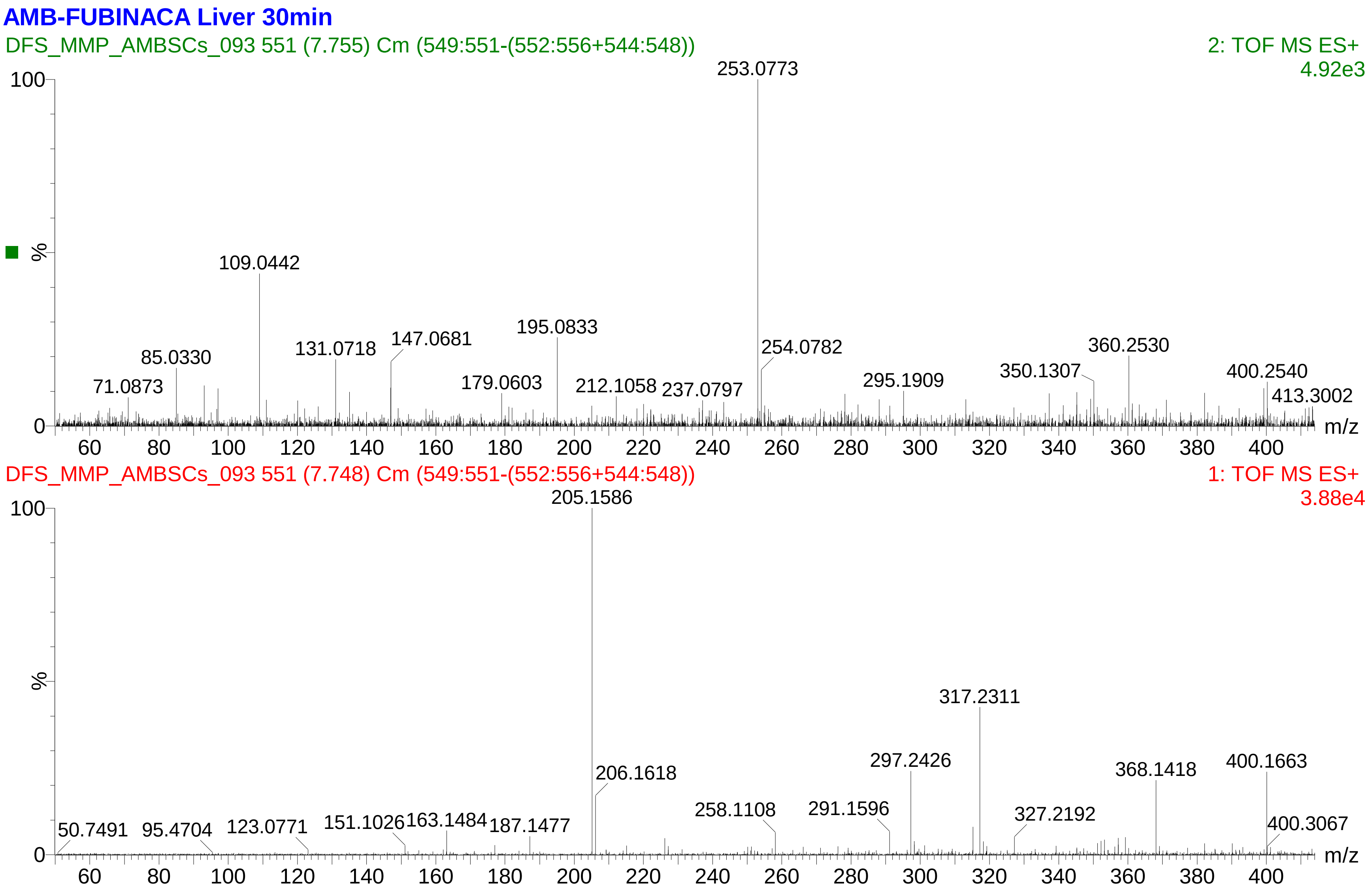

Fig. S7. Low Energy (Function 1) and High Energy (Function 2) spectra for AMB-FUBINACA M6.

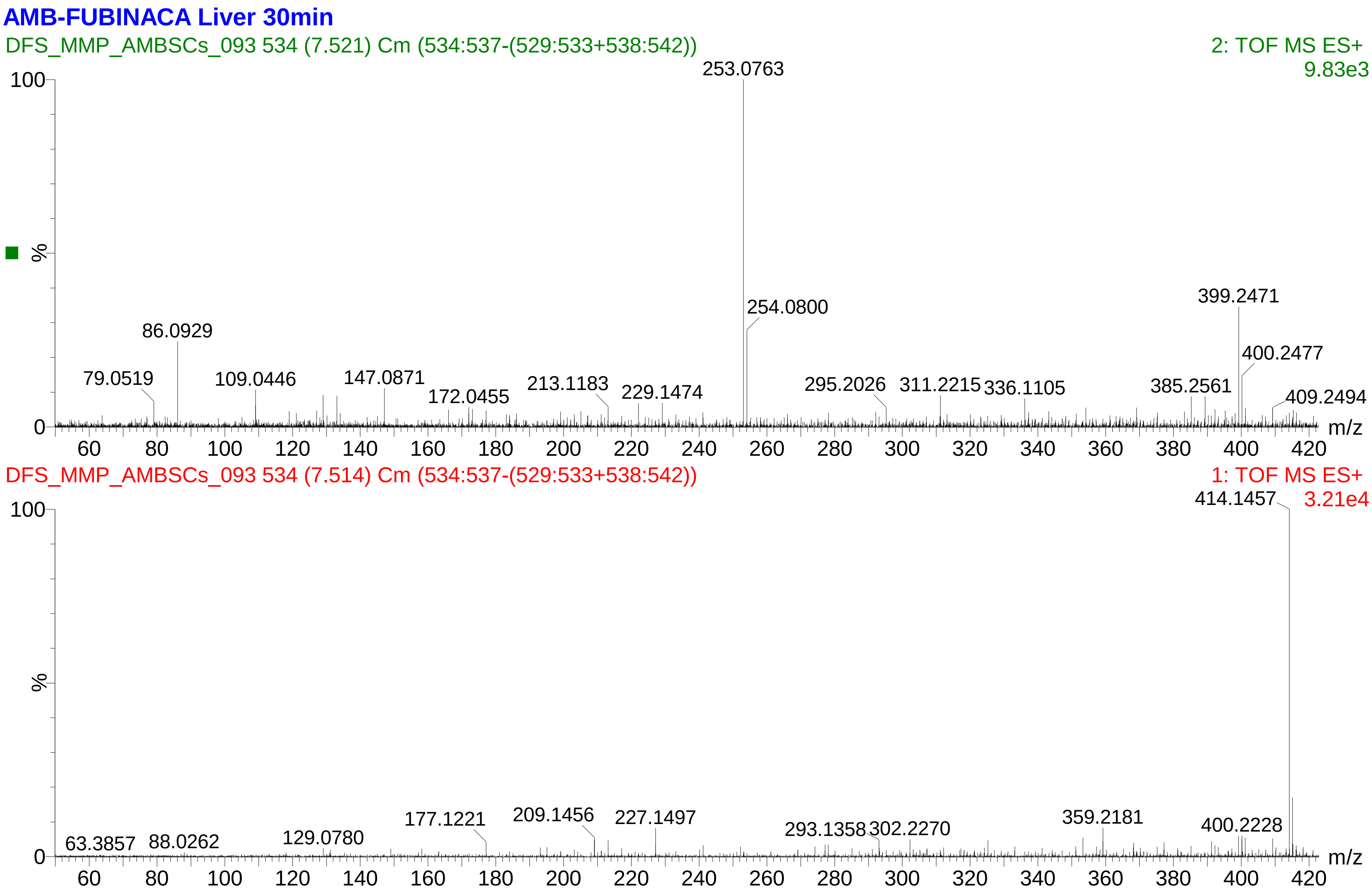

Fig. S8. Low Energy (Function 1) and High Energy (Function 2) spectra for AMB-FUBINACA M7.

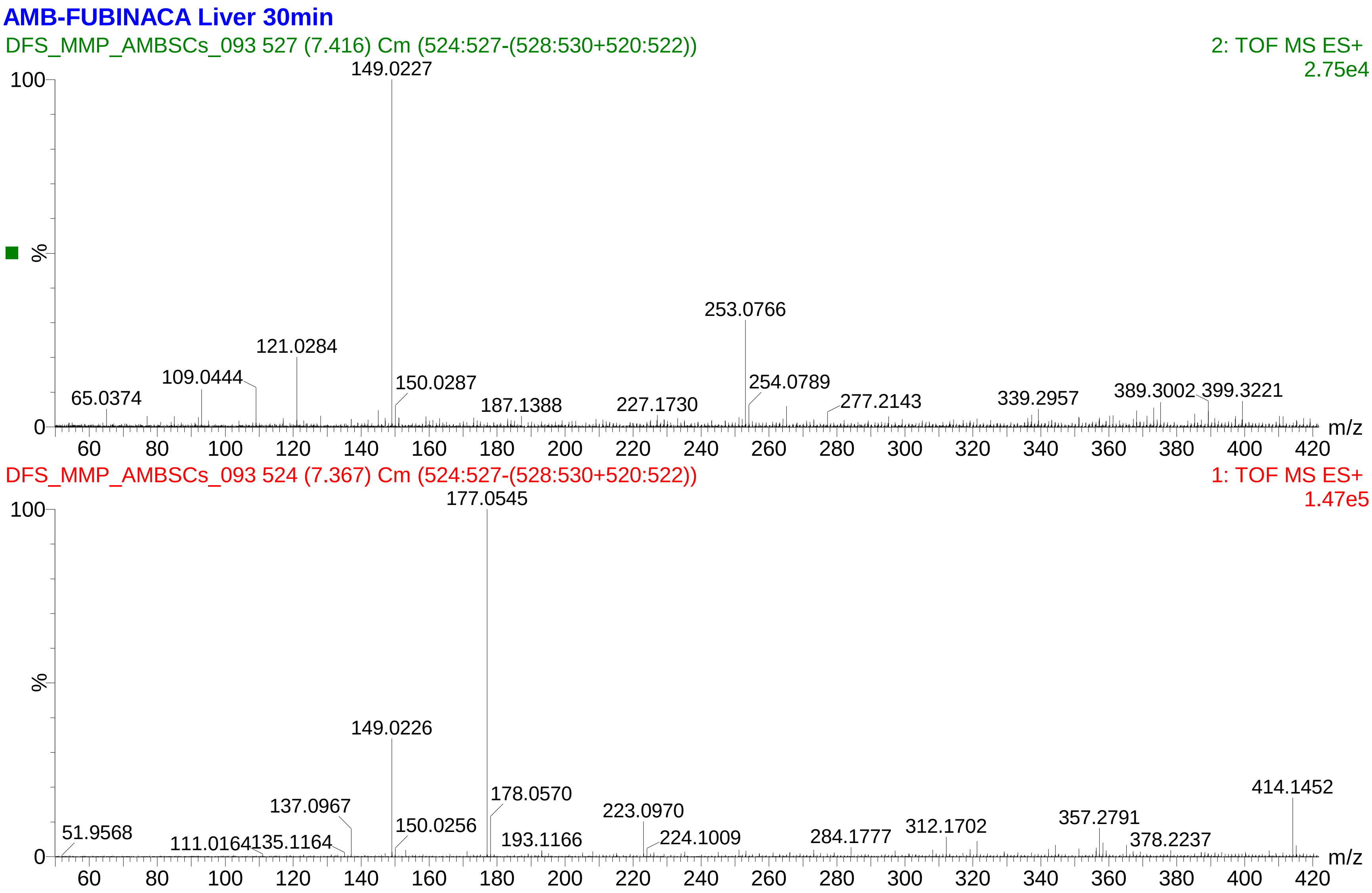

Fig. S9. Low Energy (Function 1) and High Energy (Function 2) spectra for AMB-FUBINACA M8.

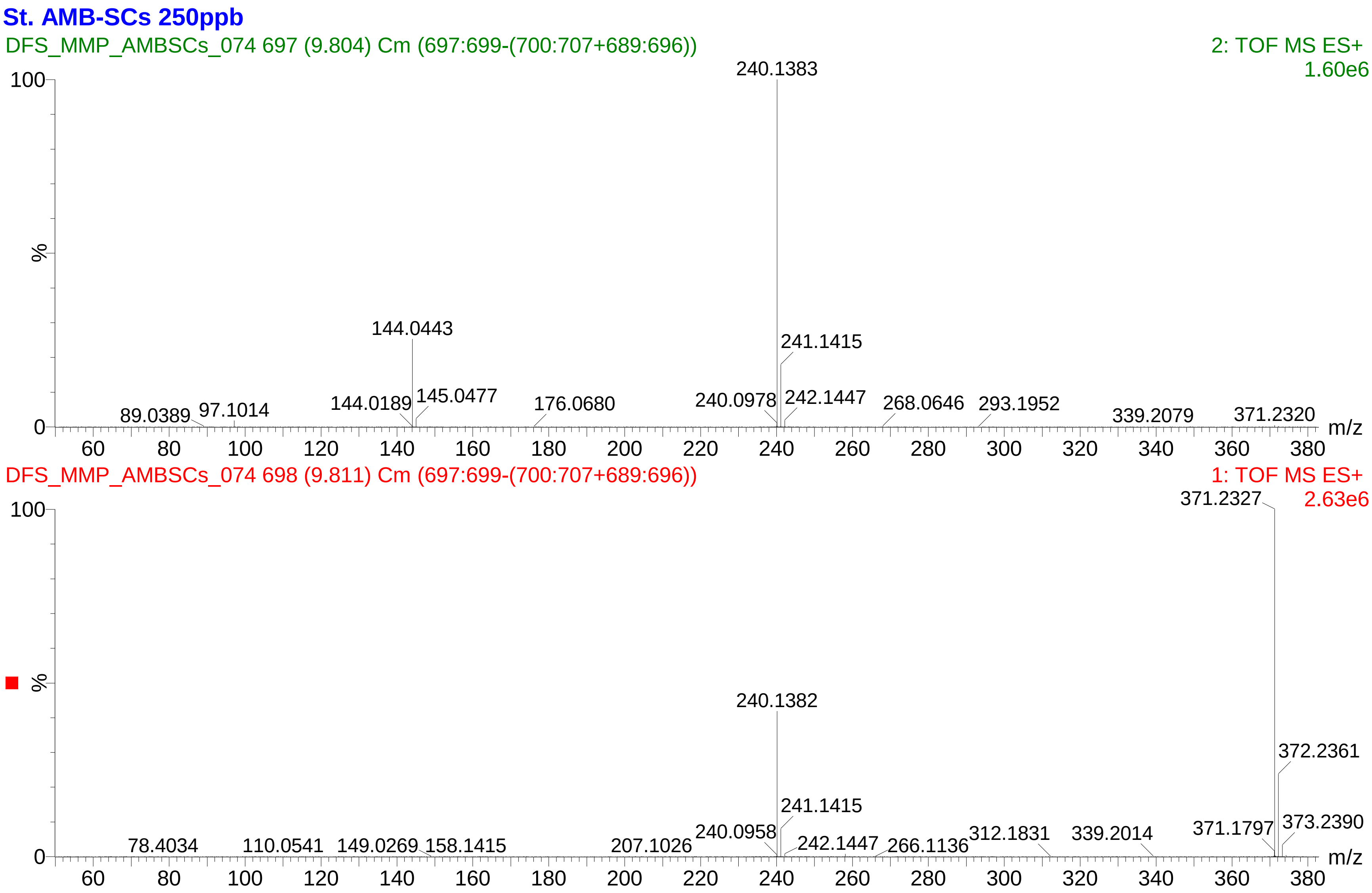

Fig. S10. Low Energy (Function 1) and High Energy (Function 2) spectra for AMB-CHMICA.

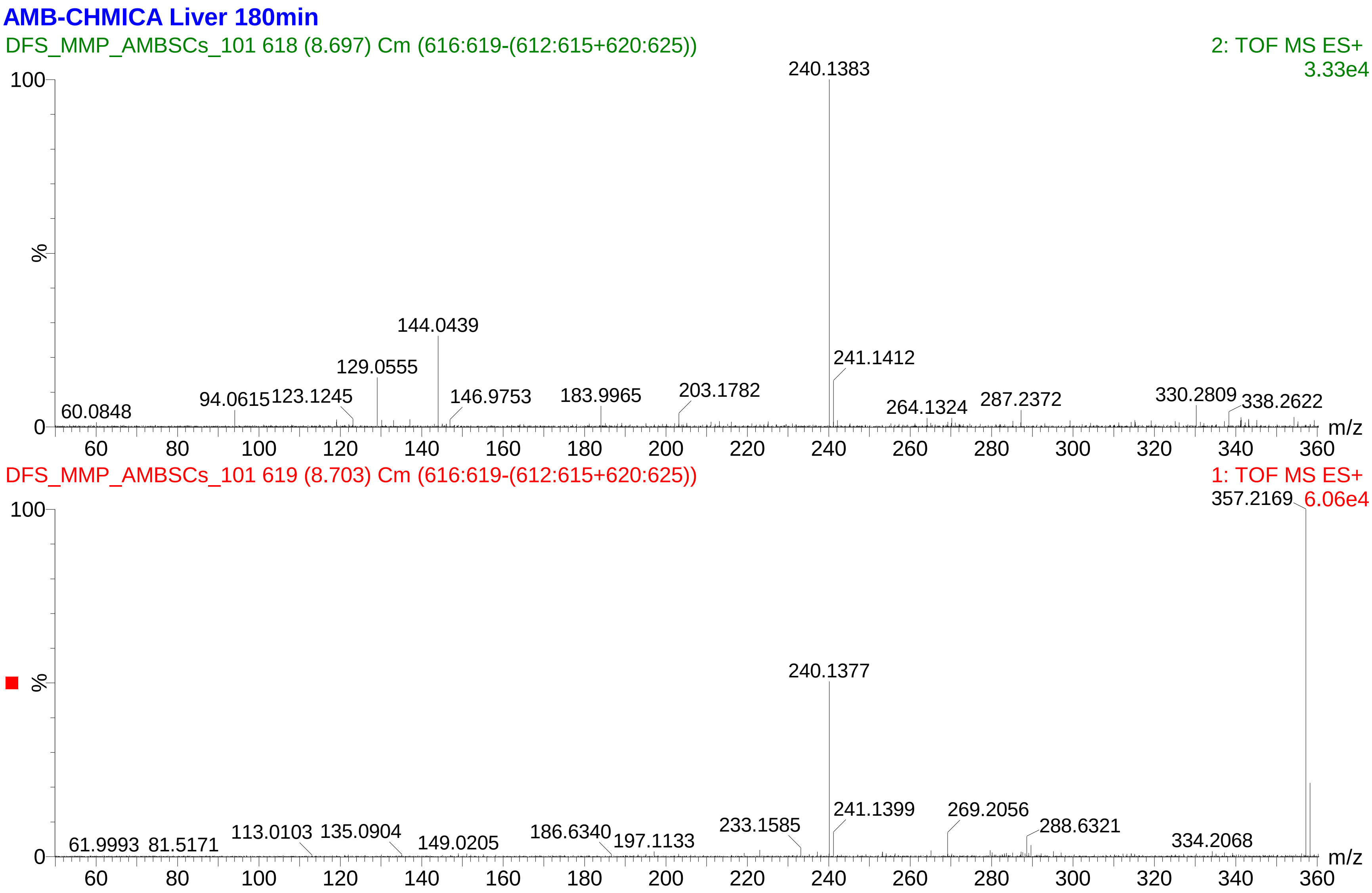

Fig. S11. Low Energy (Function 1) and High Energy (Function 2) spectra for AMB-CHMICA M1.

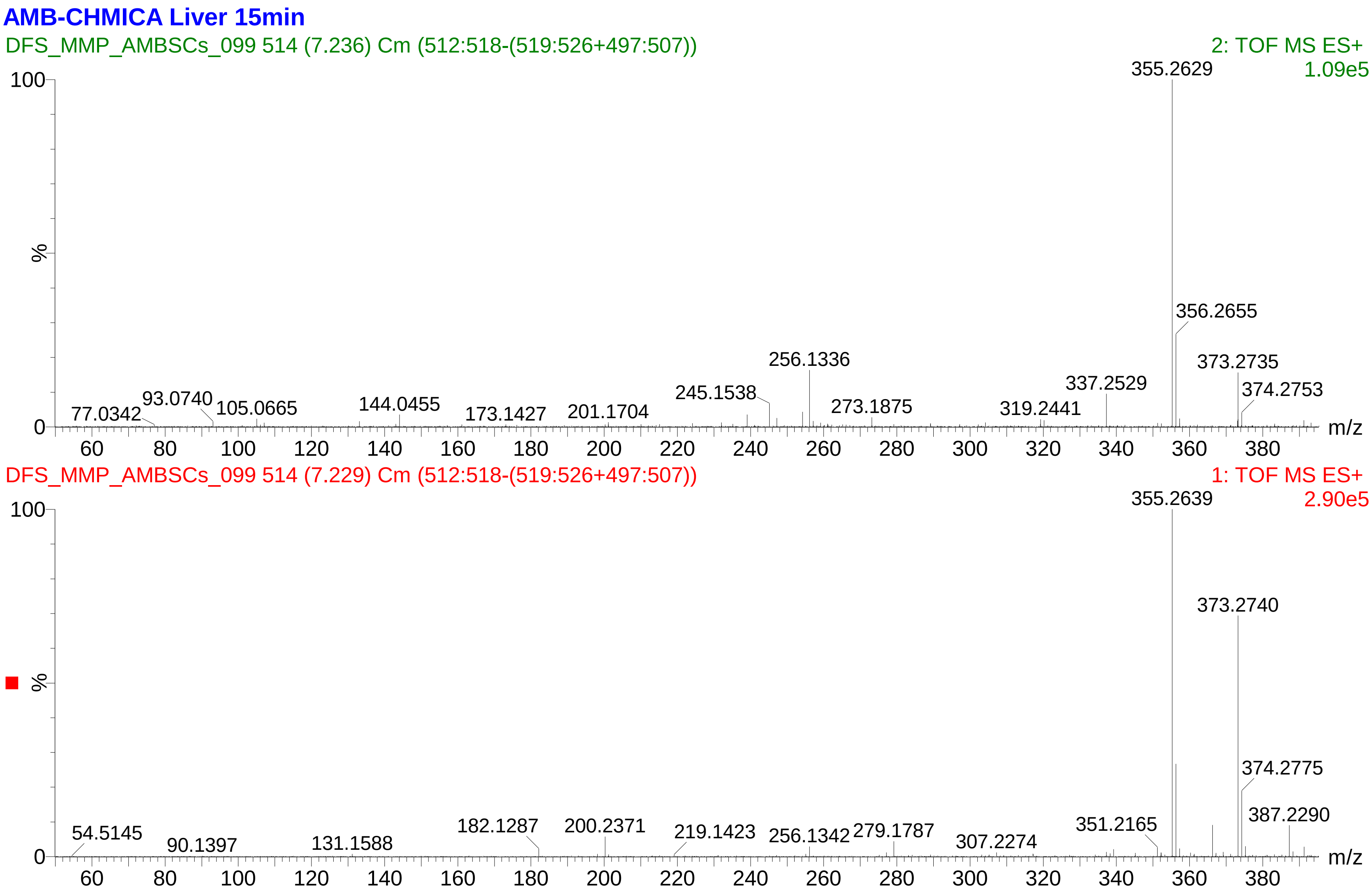

Fig. S12. Low Energy (Function 1) and High Energy (Function 2) spectra for AMB-CHMICA M2.

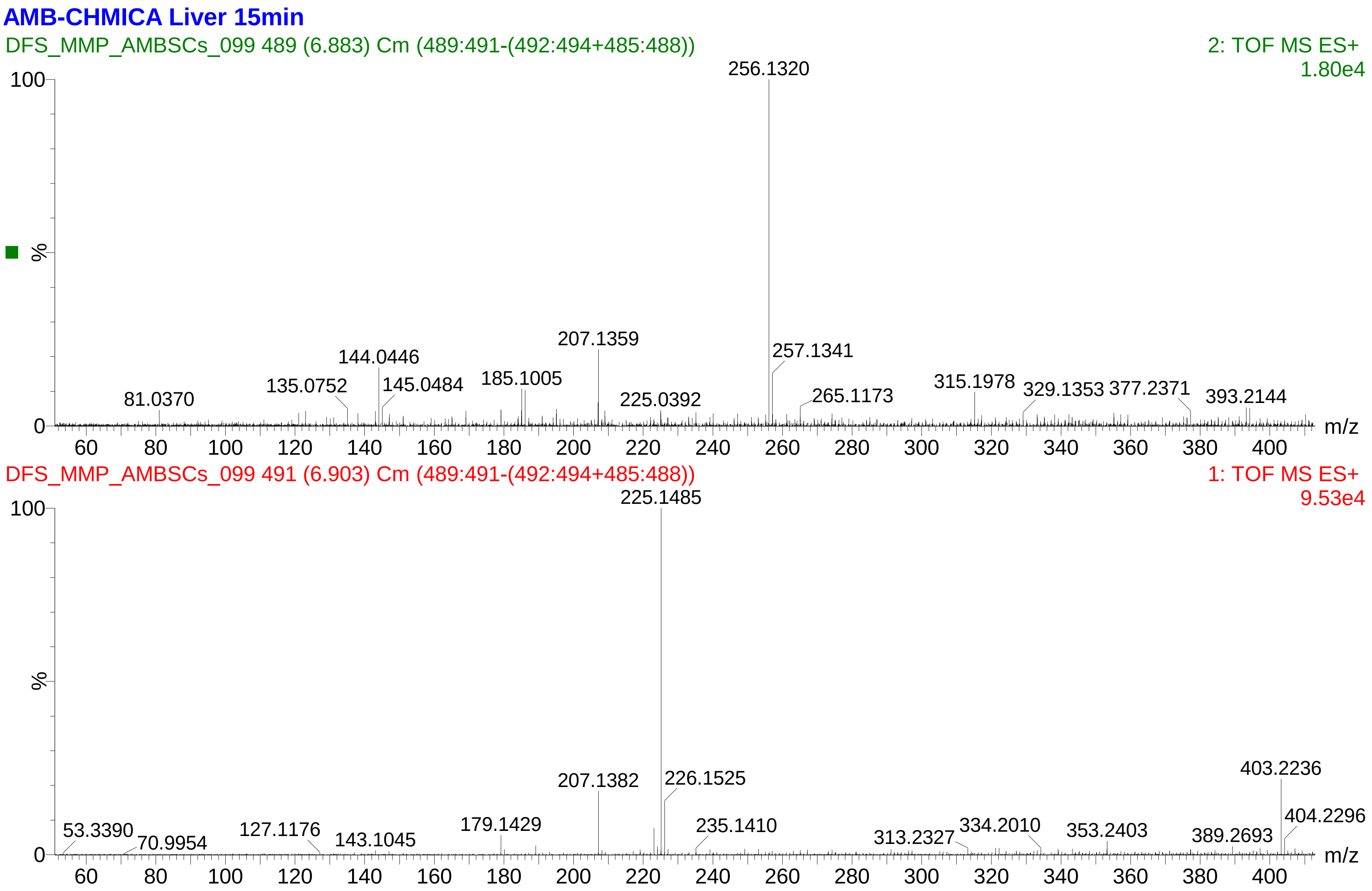

Fig. S13. Low Energy (Function 1) and High Energy (Function 2) spectra for AMB-CHMICA M3.

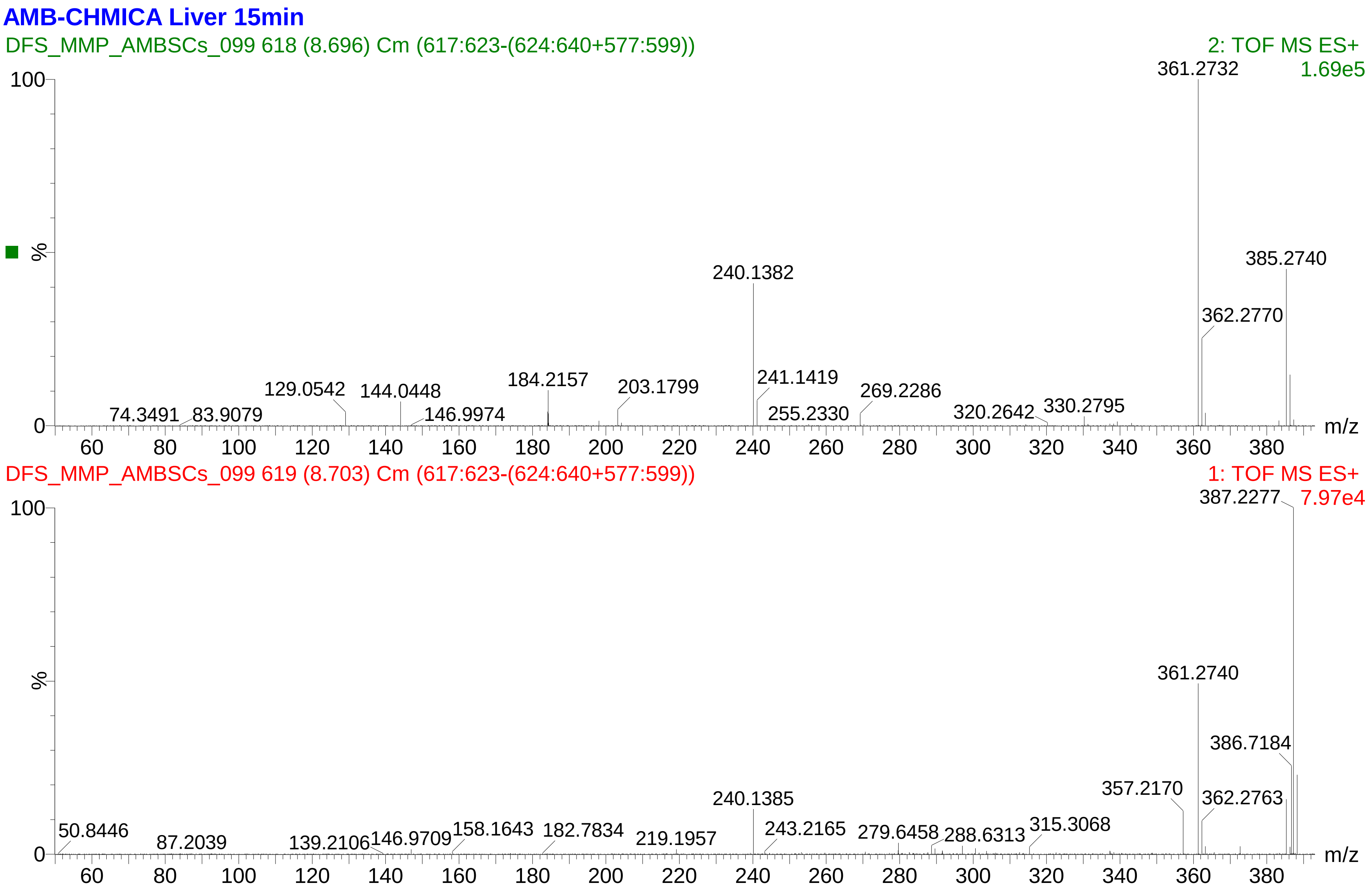

Fig. S14. Low Energy (Function 1) and High Energy (Function 2) spectra for AMB-CHMICA M4.

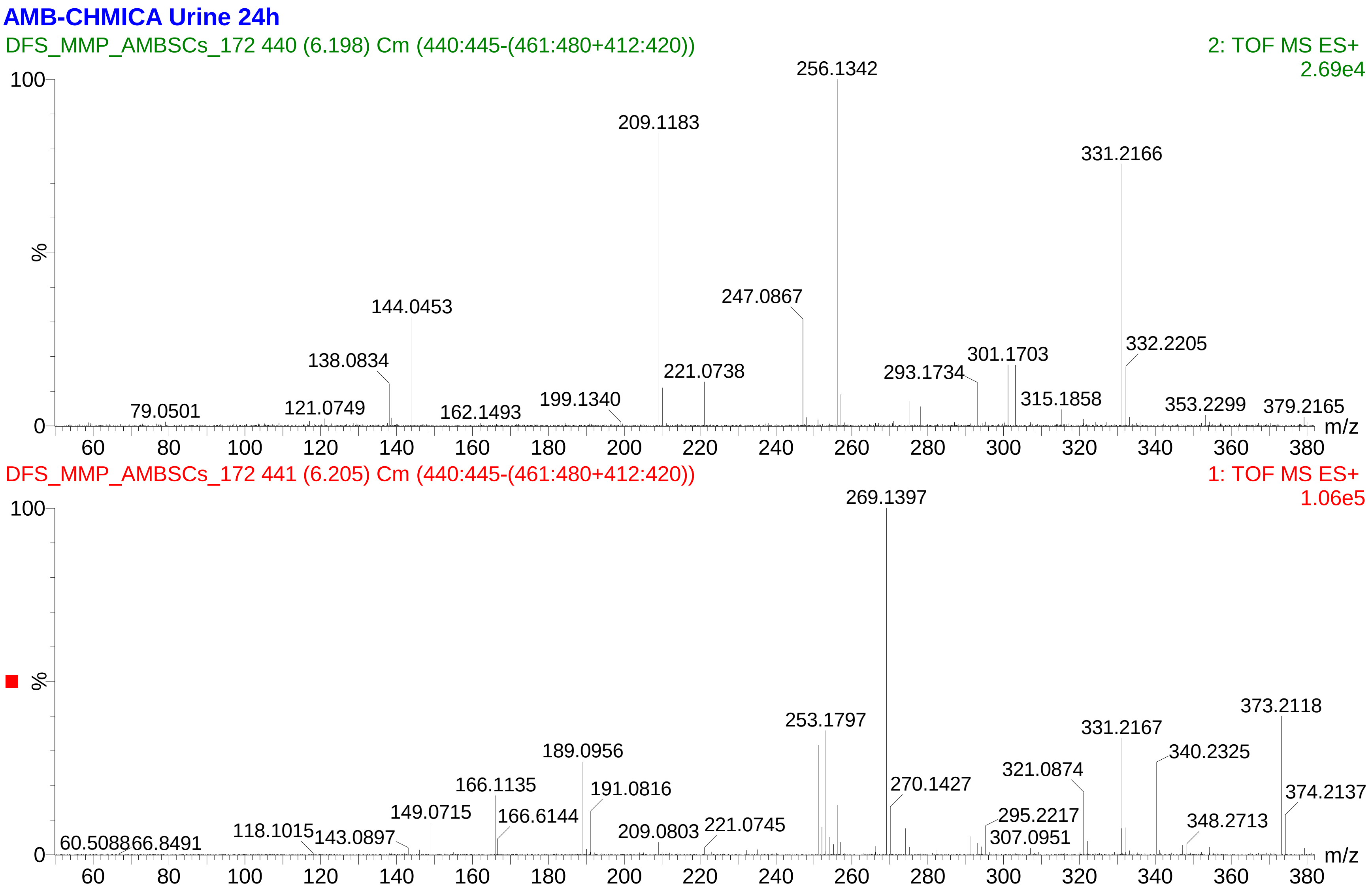

Fig. S15. Low Energy (Function 1) and High Energy (Function 2) spectra for AMB-CHMICA M5.

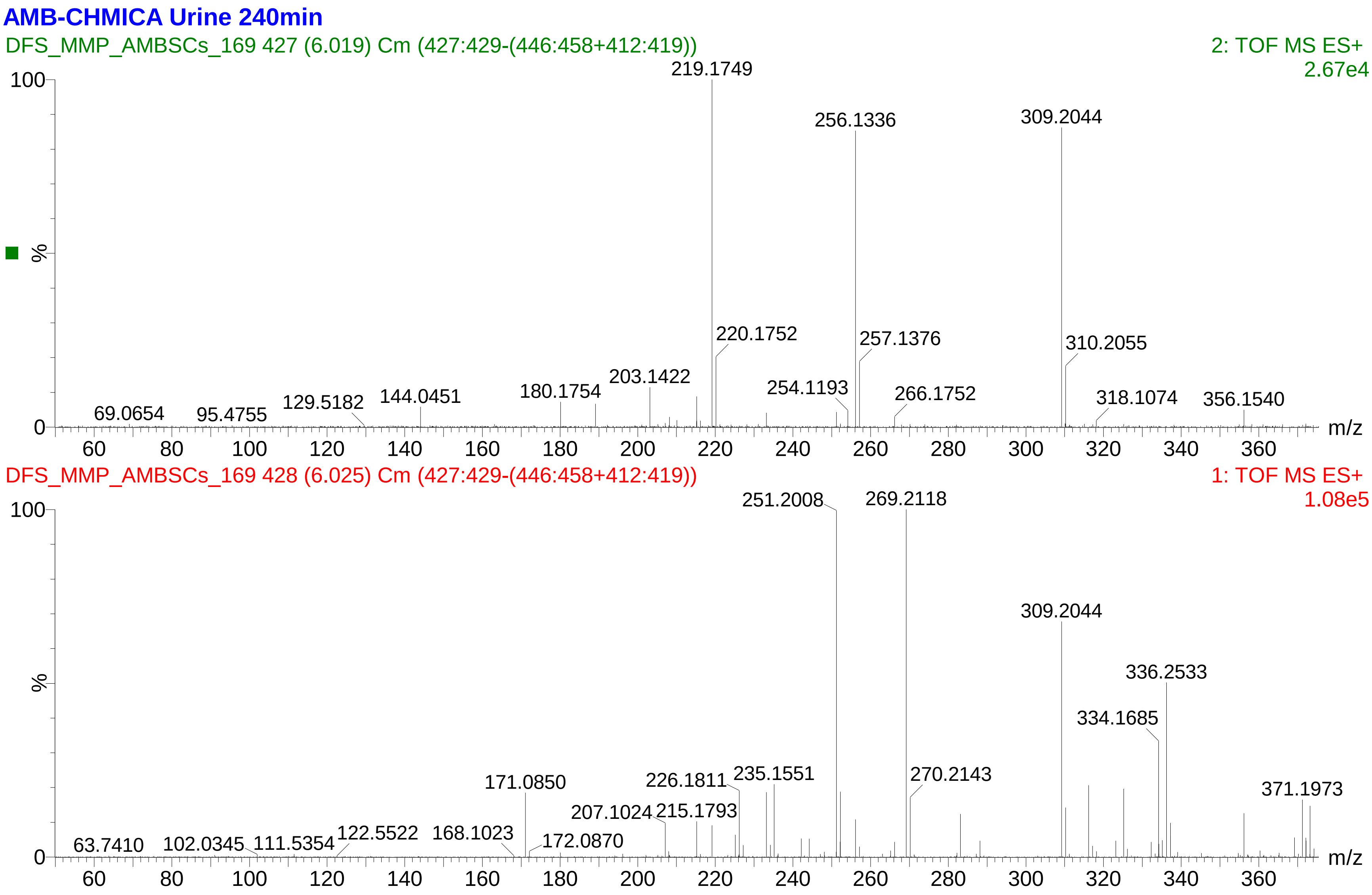

Fig. S16. Low Energy (Function 1) and High Energy (Function 2) spectra for AMB-CHMICA M6.

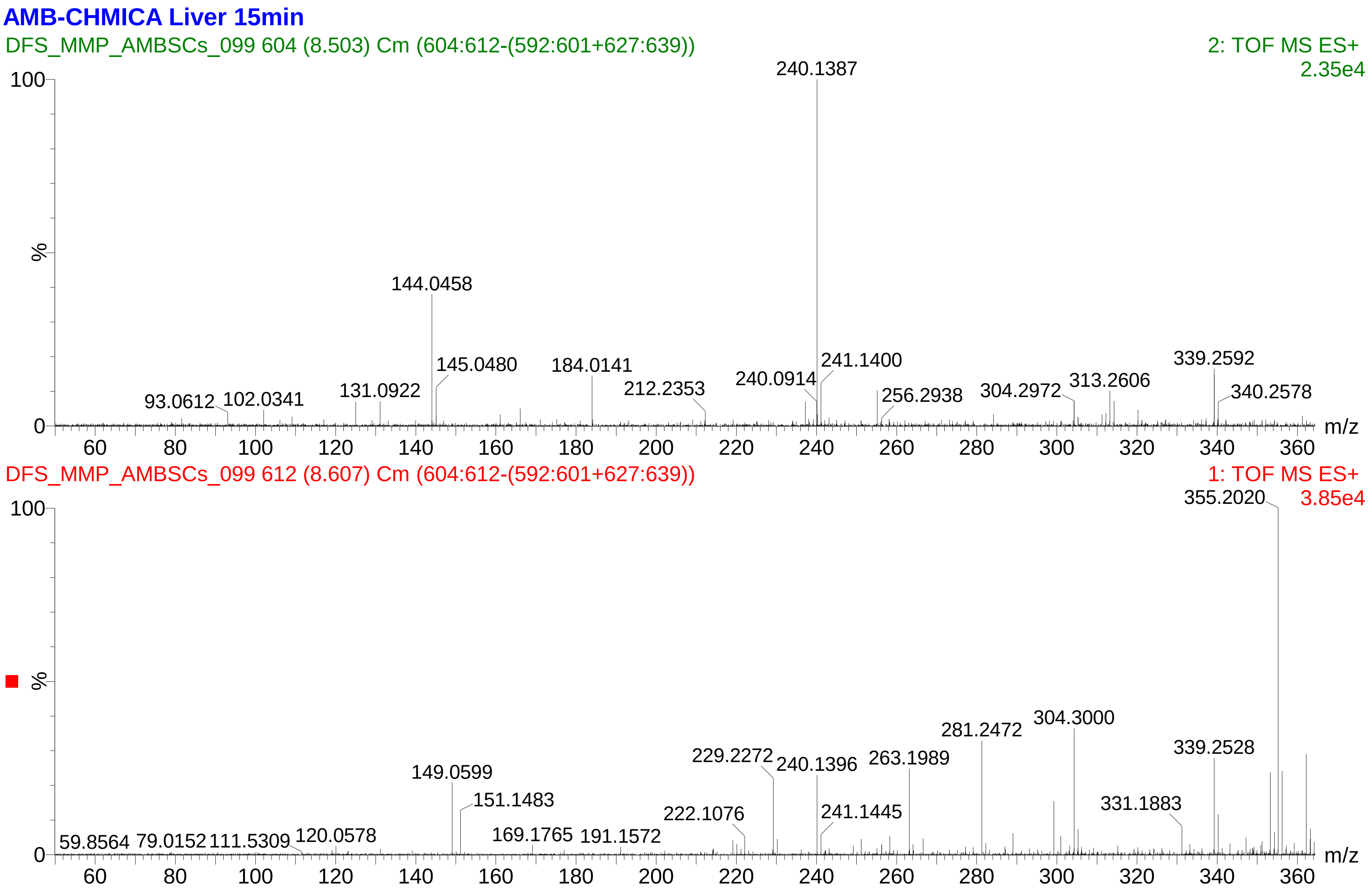

Fig. S17. Low Energy (Function 1) and High Energy (Function 2) spectra for AMB-CHMICA M7.

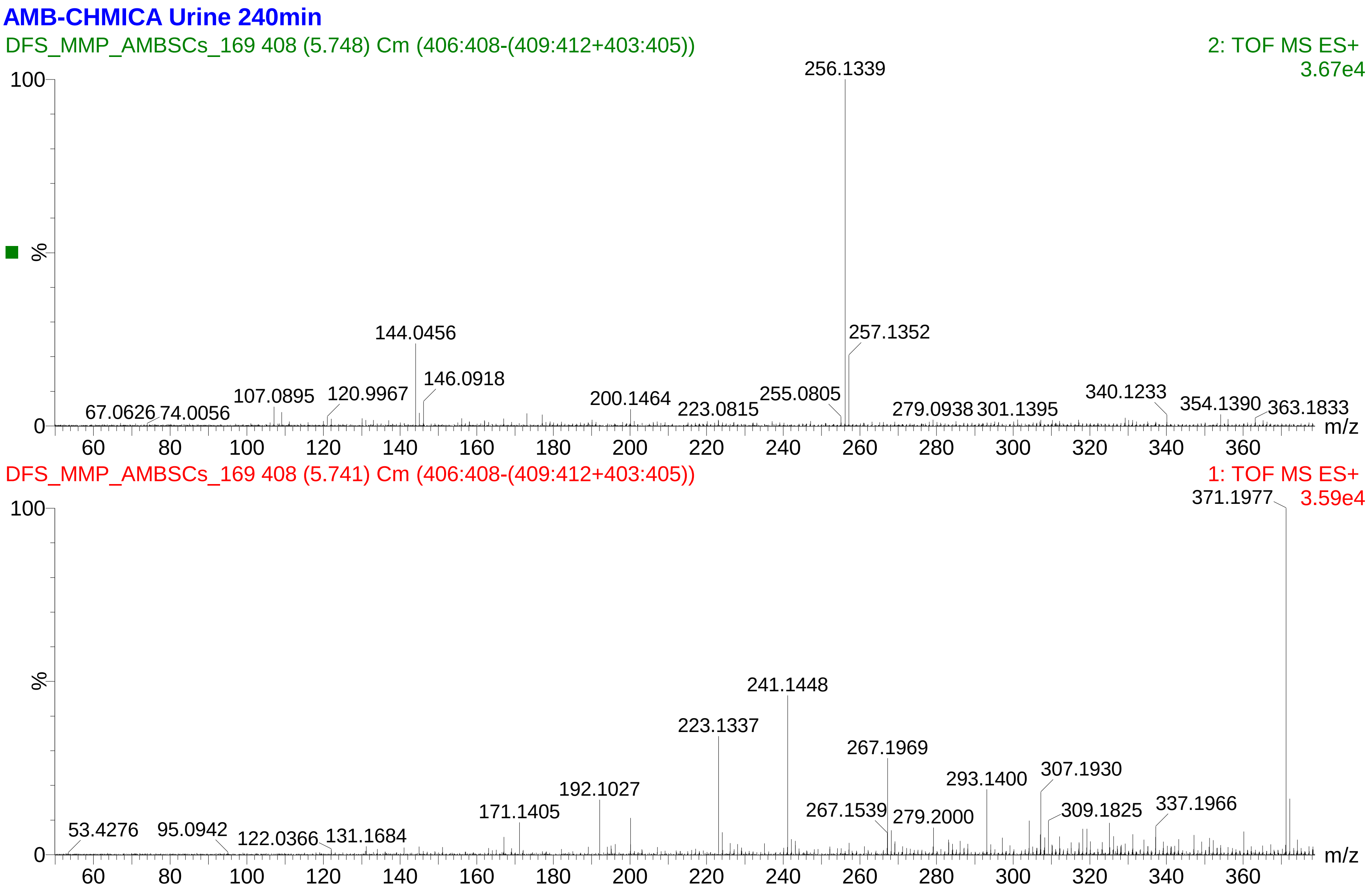

Fig. S18. Low Energy (Function 1) and High Energy (Function 2) spectra for AMB-CHMICA M8.

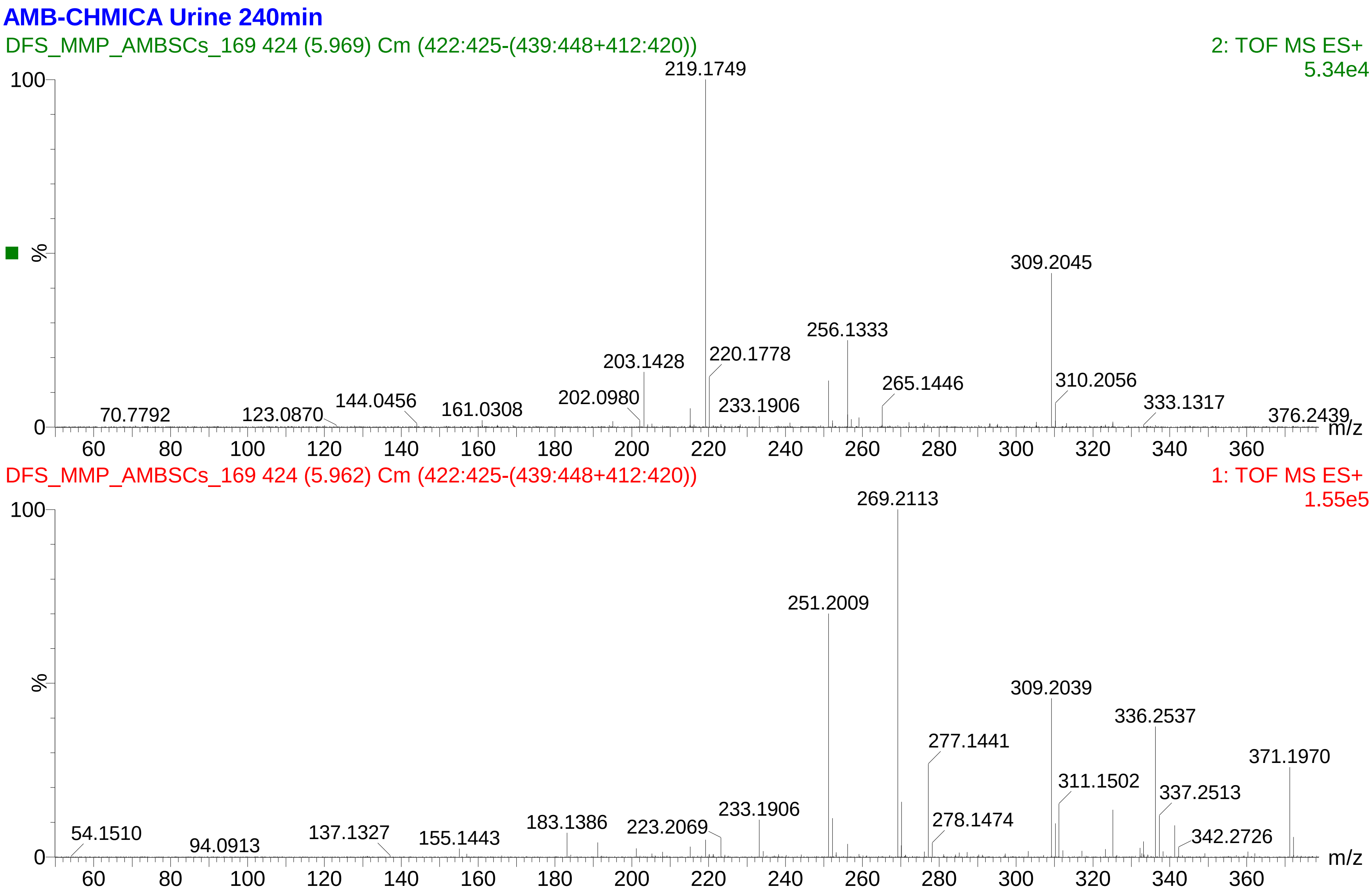

Fig. S19. Low Energy (Function 1) and High Energy (Function 2) spectra for AMB-CHMICA M9.

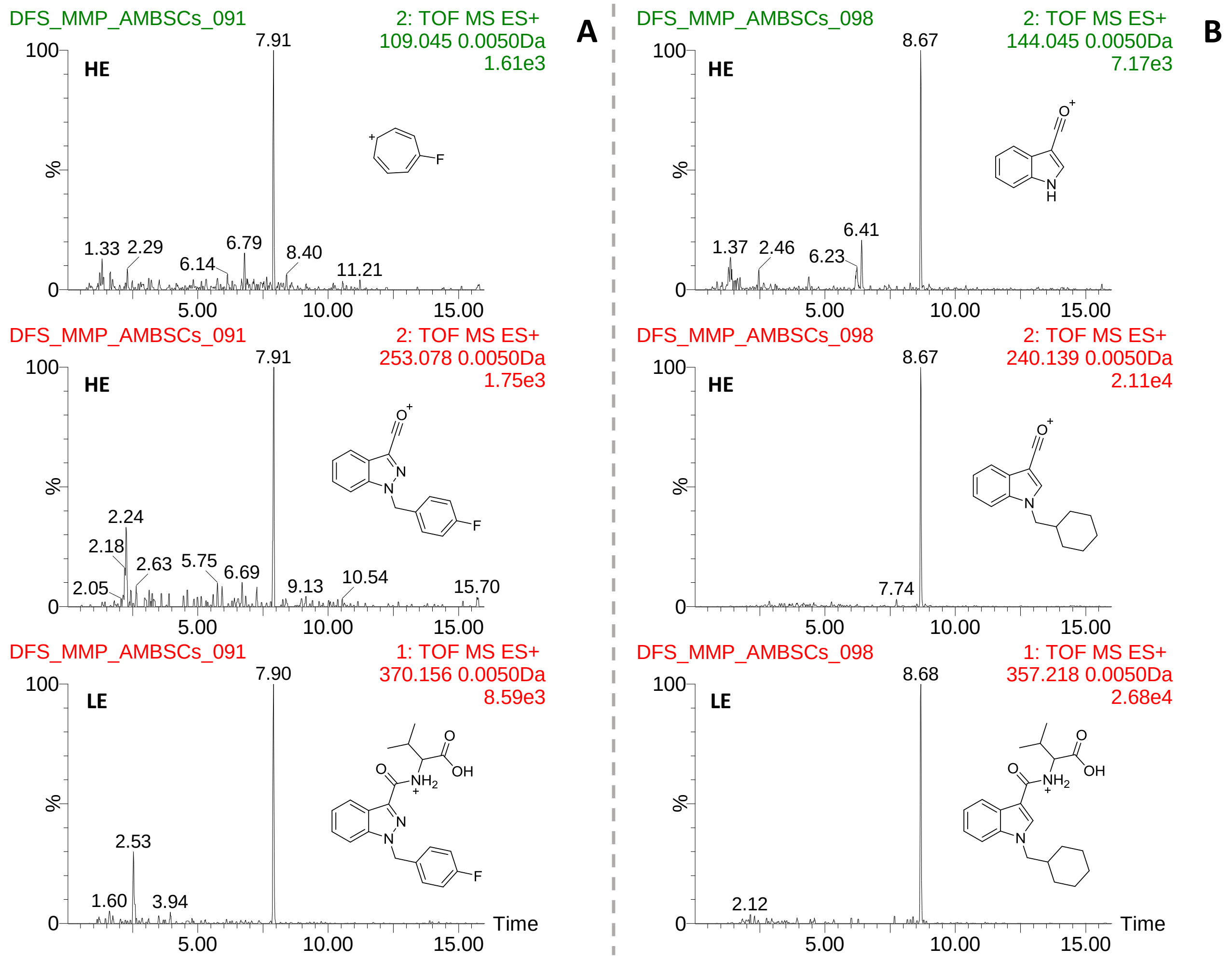

Fig. S20. Identification of the metabolites *O*-demethyl AMB-FUBINACA (**A**) and *O*-demethyl AMB-CHMICA (**B**) in liver samples collected 48 h after injection. Identification is based on chromatographic retention time and the presence of 2 accurate-mass fragment ions.

Table S1. Analytical response of AMB-FUBINACA and its detected metabolites in the different samples analysed.

Compound	Brain				Liver				Serum				Kidney				Urine					
	15 min	30 min	180 min	48 h	15 min	30 min	180 min	48 h	15 min	30 min	180 min	48 h	15 min	30 min	180 min	48 h	30 min	60 min	90 min	180 min	300 min	24 h
AMB-FUBINACA	791	219	42		77	49			162	38			548	200	37							
M1		22			1109	3519	2647	420	137	424	752	28	118	246	354					46	9	58
M2						188	98													15	46	
M3						135	108													87	48	106
M4									33	83			62	86			1204	540	243	1646	886	352
M5					55	76							139	128			2083	1204	530	3031	1483	227
M6					114	190							20	66								
M7					100	523	286			23	17			38	41			13	12	160	136	194
M8					49	405	229			23	22			42	12					39	67	42

Table S2. Analytical response of AMB-CHMICA and its detected metabolites in the different samples analysed.

Compound	Brain				Liver				Serum				Kidney				Urine							
	15 min	30 min	180 min	48 h	15 min	30 min	180 min	48 h	15 min	30 min	180 min	48 h	15 min	30 min	180 min	48 h	30 min	60 min	90 min	120 min	240 min	300 min	360 min	24 h
AMB-CHMICA	214	205			7928	6735	43		394	576	34		313	478										
M1					325	441	1000	1252			45		275	191										254
M2					591	268			46	70														
M3					490	387																		
M4	13	31			1433	1083				36				154										
M5																				79	127	261	187	806
M6																			164	348	495	430	362	134
M7		79			588	880	65		79	244	53		129	258	50									
M8						132								117				58	318	594	712	724	600	192
M9																			169	439	665	647	515	231
