# Supplementary material for: In-depth comparison of the metabolic and pharmacokinetic behaviour of the structurally related synthetic cannabinoids AMB-FUBINACA and AMB-CHMICA in rats
Source: Commun Biol. 2022 Feb 24;5:161. doi: 10.1038/s42003-022-03113-5 (PMC8873228; doi:10.1038/s42003-022-03113-5)
Supplement: Supplementary file 2 — Reporting Summary [file 42003_2022_3113_MOESM2_ESM.pdf]

nature portfolio

Corresponding author(s): María Ibáñez

Last updated by author(s): Dec 22, 2021

Reporting Summary

Nature Portfolio wishes to improve the reproducibility of the work that we publish. This form provides structure for consistency and transparency in reporting. For further information on Nature Portfolio policies, see our Editorial Policies and the Editorial Policy Checklist.

Statistics

For all statistical analyses, confirm that the following items are present in the figure legend, table legend, main text, or Methods section.

n/a	Confirmed	
☒	☐	The exact sample size (*n*) for each experimental group/condition, given as a discrete number and unit of measurement
☒	☐	A statement on whether measurements were taken from distinct samples or whether the same sample was measured repeatedly
☒	☐	The statistical test(s) used AND whether they are one- or two-sided<br>*Only common tests should be described solely by name; describe more complex techniques in the Methods section.*
☒	☐	A description of all covariates tested
☒	☐	A description of any assumptions or corrections, such as tests of normality and adjustment for multiple comparisons
☒	☐	A full description of the statistical parameters including central tendency (e.g. means) or other basic estimates (e.g. regression coefficient) AND variation (e.g. standard deviation) or associated estimates of uncertainty (e.g. confidence intervals)
☒	☐	For null hypothesis testing, the test statistic (e.g. *F*, *t*, *r*) with confidence intervals, effect sizes, degrees of freedom and *P* value noted<br>*Give P values as exact values whenever suitable.*
☒	☐	For Bayesian analysis, information on the choice of priors and Markov chain Monte Carlo settings
☒	☐	For hierarchical and complex designs, identification of the appropriate level for tests and full reporting of outcomes
☒	☐	Estimates of effect sizes (e.g. Cohen's *d*, Pearson's *r*), indicating how they were calculated

Our web collection on statistics for biologists contains articles on many of the points above.

Software and code

Policy information about availability of computer code

Data collection	MassLynx v4.0
Data analysis	MassLynx v4.0, TargetLynx XS, ChromaLynx XS, MetaboLynx XS, ChemDraw v20.1, Microsoft Office 2016.

For manuscripts utilizing custom algorithms or software that are central to the research but not yet described in published literature, software must be made available to editors and reviewers. We strongly encourage code deposition in a community repository (e.g. GitHub). See the Nature Portfolio guidelines for submitting code & software for further information.

Data

Policy information about availability of data

All manuscripts must include a data availability statement. This statement should provide the following information, where applicable:

- Accession codes, unique identifiers, or web links for publicly available datasets
- A description of any restrictions on data availability
- For clinical datasets or third party data, please ensure that the statement adheres to our policy

Authors can confirm that all relevant data are included in the article and/or its supplementary information files.

Field-specific reporting

Life sciences study design

All studies must disclose on these points even when the disclosure is negative.

Sample size	8 animals were used, as this number is enough as no statistical analysis has been made.
Data exclusions	No data was excluded.
Replication	No replicates were performed.
Randomization	No randomization was needed for this study, as the aim was to elucidate metabolites of known synthetic cannabinoids, as well as their pharmacokinetics.
Blinding	Blinding is not relevant in this study, as the aim was to elucidate metabolites of known synthetic cannabinoids, as well as their pharmacokinetics.

Reporting for specific materials, systems and methods

We require information from authors about some types of materials, experimental systems and methods used in many studies. Here, indicate whether each material, system or method listed is relevant to your study. If you are not sure if a list item applies to your research, read the appropriate section before selecting a response.

Materials & experimental systems

n/a	Involved in the study	
☒	☐	Antibodies
☒	☐	Eukaryotic cell lines
☒	☐	Palaeontology and archaeology
☐	☒	Animals and other organisms
☒	☐	Human research participants
☒	☐	Clinical data
☒	☐	Dual use research of concern

Methods

n/a	Involved in the study	
☒	☐	ChIP-seq
☒	☐	Flow cytometry
☒	☐	MRI-based neuroimaging

Animals and other organisms

Policy information about studies involving animals; ARRIVE guidelines recommended for reporting animal research

Laboratory animals	Sprague-Dawley rats (8 weeks old, weighing between 385 and 425 g)
Wild animals	The study did not involvedwild animals.
Field-collected samples	Animals were housed individually in polypropylene plastic cages under controlled temperature (24 ±2 ºC) and lighting conditions (12 h:12 h; lights ON at 8 AM), with ad libitum access to food and water. Before SCRA injection, animals were handled and habituated to the experimental room for 1 week.
Ethics oversight	All the experiments were approved by the Committee of Ethics and Animal Experimentation of Universitat Jaume I (2019/VSC/PEA/048) and treated throughout according to the European Union Council Directive of June 3rd, 2010 (6106/1/10 REV1).

Note that full information on the approval of the study protocol must also be provided in the manuscript.
